# Characterization of strain-specific *Bacillus cereus* swimming motility and flagella by means of specific antibodies

**DOI:** 10.1371/journal.pone.0265425

**Published:** 2022-03-17

**Authors:** Valerie Schwenk, Richard Dietrich, Andreas Klingl, Erwin Märtlbauer, Nadja Jessberger

**Affiliations:** 1 Department of Veterinary Sciences, Faculty of Veterinary Medicine, Ludwig-Maximilians-Universität München, Oberschleißheim, Germany; 2 Department of Biology I, Plant Development and Electron Microscopy, Biocenter Ludwig-Maximilians Universität München, Planegg-Martinsried, München, Germany; 3 Institute for Food Quality and Food Safety, University of Veterinary Medicine Hannover, Hannover, Germany; Universita degli Studi di Pavia, ITALY

## Abstract

One of the multiple factors determining the onset of the diarrhoeal disease caused by enteropathogenic *Bacillus cereus* is the ability of the bacteria to actively move towards the site of infection. This ability depends on flagella, but it also varies widely between different strains. To gain more insights into these strain-specific variations, polyclonal rabbit antisera as well as monoclonal antibodies (mAbs) were generated in this study, which detected recombinant and natural *B*. *cereus* flagellin proteins in Western blots as well as in enzyme immunoassays (EIAs). Based on mAb 1A11 and HRP-labelled rabbit serum, a highly specific sandwich EIA was developed. Overall, it could be shown that strain-specific swimming motility correlates with the presence of flagella/flagellin titres obtained in EIAs. Interestingly, mAb 1A11, recognizing an epitope in the N-terminal region of the flagellin protein, proved to inhibit bacterial swimming motility, while the rabbit serum rather decreased growth of selected *B*. *cereus* strains. Altogether, powerful tools enabling the in-depth characterization of the strain-specific variations in *B*. *cereus* swimming motility were developed.

## Introduction

Bacterial flagella have been in the focus of scientific research for decades. Enormous progress has been made in understanding flagellar assembly, the axial structure, as well as the mechanism of motor rotation [1–4]. The flagellum is a filamentous organelle consisting of more than 25 different proteins and can be divided into three structural parts: the basal body containing the ion motive force-powered motor, the long helical filament, which acts as propeller, and the hook connecting the basal body and the filament [5, 6]. The flagellar filament consists of approximately 20,000 copies of the protein monomer flagellin [1, 4]. In *Escherichia coli*, one of the best-understood model organisms regarding flagella, the flagellin protein is designated FliC. Other bacterial species possess multiple flagellins, of which, however, many are not necessarily required for filament formation [3, 7]. For instance, in *Bacillus subtilis*, two homologs of the flagellin gene are found, namely *yvzB* and *hag*, but only the latter is required for flagellar assembly and motility [2]. Due to a different structure of the Hag protein, the diameter of the *B*. *subtilis* filament is much smaller than that of for instance the *Salmonella* filament [6]. *Bacillus thuringiensis* possesses *fliC* and *hag* homologues, and two or even three copies of *hag* were found in some strains [8–10]. *Bacillus cereus* type strain ATCC 14579 has four flagellin genes, of which three are highly similar and the corresponding transcripts and proteins have been detected [11, 12] (compare also points A and B in S2 Fig). Tagawa showed that the flagellum consists of equal amounts of these three 34, 32 and 31 kDa flagellin proteins, but also emphasized that ATCC 14579 is atypical among *B*. *cereus* strains bearing multiple genes that encode flagellin subunits. The genomes of other investigated *B*. *cereus* strains contain two flagellin gene homologues, one coding for a flagellin subunit, and the other similar to the non-expressed fourth gene BC1656 of strain ATCC 14579 [9].

The best understood function of the bacterial flagellum is swimming motility in liquid environments. Bacterial chemoreceptors sense chemical gradients of attractants and repellents, and intracellular signalling pathways trigger changes in flagellar rotation direction. Thus, the cell is able to switch between straight swimming and tumbling, enabling movement towards more advantageous environments [1–3]. Besides that, bacteria can move collectively over solid surfaces via flagella-driven swarming motility. For this, the flagellum is also believed to act as a sensor, as contact with a more solid surface slows the flagellum down [13]. Swarm cells often change to a hyper-flagellated and elongated morphology [2, 5]. Chemotaxis and motility are also required for biofilm formation [14, 15]. Biofilms consist of cell aggregates attached to solid surfaces, which are surrounded by an extracellular polymeric matrix [1]. Approximately 65% of all bacterial infections are associated with biofilms [1, 16]. Chemotaxis, swimming and swarming motility and thus flagella are described as crucial virulence factors for pathogenic bacteria by enhancing antibiotic resistance [13, 17], facilitating movement towards infection sites and adherence to target host cells, providing colonization or invasion and infection of the epithelial surface, promoting bacteria-host interactions, and triggering inflammation [1, 18–21]. Flagella are further associated with virulence-related protein secretion [18].

Flagella also play an important role in the course of food-associated infections with enteropathogenic *B*. *cereus* resulting in abdominal cramps and diarrhoea, which we recently summarized [22, 23]. While some members of the *B*. *cereus* group (*B*. *cereus sensu lato*) are described as non-motile, the majority of strains of *B*. *cereus sensu stricto*, *B*. *thuringiensis* and *B*. *cytotoxicus* is motile [24, 25]. This was also seen when 20 enteropathogenic and apathogenic *B*. *cereus* strains were compared on CGY soft-agar at 30 and 37°C. Results suggested that motility of *B*. *cereus* is temperature-dependent to some extent, but also highly strain-specific [26]. In earlier studies, clinical strains including those isolated following food poisoning were generally more motile than non-pathogenic strains [27, 28]. Swimming and swarming motility were also correlated with secretion of important virulence factors such as haemolysin BL, phospholipase C, sphingomyelinase or cytotoxin K, strongly connecting motility and pathogenicity [12, 28–34]. A connection between motility, biofilm formation and virulence in the *B*. *cereus* group has also been made via the ubiquitous transcriptional repressor MogR. Interestingly, genes involved in chemotaxis and motility as well as flagellar gene regulation in *B*. *cereus* seems to be more closely related to *Listeria monocytogenes* than to *B*. *subtilis* [24]. Just recently, it has been shown that *B*. *cereus* flagellin and the host-cell-surface-localized glycosphingolipid Gb3 interact, promoting adhesion and thus, virulence and lethality [35].

The goal of this study was to better understand the highly strain-specific variations in swimming motility among enteropathogenic *B*. *cereus*. For that, antibody-based tools for specific detection of *B*. *cereus* flagellin were developed, which can also be used for precise inhibition of bacterial growth or motility.

## Materials and methods

### Ethics statement

Immunizations of rabbits and mice for generating antisera and monoclonal antibodies were conducted in compliance with the German Law for Protection of Animals. Study permissions were obtained by the Government of Upper Bavaria (permit numbers ROB-55.2-2532.Vet_03-17-110 and 55.2-1-54-2532.0-47-2015, respectively).

### Bacterial strains and culture conditions

In this study, a set of 20 previously well-characterized, enteropathogenic and apathogenic *B*. *cereus* strains was investigated [26, 36, 37]. Additionally, a *nheABC* deletion mutant of strain F837/76 (DSM 4222) was used [38] as well as *B*. *cereus* type strain ATCC 14579. *B*. *thuringiensis* MHI 3186, 3240 and MHI 3241 [39], *B*. *weihenstephanensis* MHI 3351, *B*. *pseudomycoides* MHI 3346 (DSM 12442), *B*. *subtilis* MHI 255 (ATCC 2109), MHI 256 (ATCC 6633), MHI 3192 (DSM 3256) and MHI 3193 (DSM 10), *B*. *licheniformis* MHI 3248, *B*. *amyloliquefaciens* MHI 3352 (DSM 7), *B*. *pumilus* MHI 3354 (DSM 27), *L*. *monocytogenes* MHI 1126 (SLCC 5581), *E*. *coli* BL21 (DE3), and an in-house Salmonella strain were applied as controls in Western blots and enzyme immunoassays. *Bacillus* strains were grown in CGY medium (casein-glucose-yeast; 2% casein hydrolysate, Oxoid, 0.6% yeast extract, Oxoid, 0.2% ammonium sulphate, 1.4% K_2_HPO_4_, Sigma-Aldrich, 0.6% KH_2_PO_4_, Sigma-Aldrich, 0.1% sodium citrate dihydrate, Merck, 0.2% MgSO_4_ x 7H_2_O, Sigma-Aldrich; Merck and Thermo Scientific, Germany) with 1% glucose for 6 h at 37°C (*B*. *weihenstephanensis* 32°C) and 200 rpm, *Salmonella* and *E*. *coli* in LB (1% tryptone, 1% NaCl, 0.5% yeast extract, Sigma-Aldrich; Merck, Germany) at 37°C, and *L*. *monocytogenes* in BHI (brain heart infusion, Oxoid; Thermo Scientific, Germany) at 30°C. To better compare all *Bacillus* samples, they were set to OD_600_ = 9, before 1 ml of each bacterial sample was heated for 15 min at 95°C and centrifuged (12 min, 8.000 g, 4°C). The supernatant was kept at -20°C until further usage. For RNA preparation, *B*. *cereus* strains were grown in CGY medium for 3 h, before 5 ml samples were centrifuged (10 min, 3500 g, 4°C) and the cell pellets were stored at -80°C. For immunofluorescence, CGY medium was inoculated with *B*. *cereus* overnight cultures to OD_600_ = 0.2. At OD_600_ = 1, cells were harvested by centrifugation (1 ml, 3 min, 8.000 g, room temperature). Recombinant flagellin proteins were overexpressed in *E*. *coli* BL21 (DE3), which was grown in LB medium containing 100 μg/ml ampicillin.

### Cloning, overexpression and purification of recombinant flagellin proteins

The *flaA* gene of *B*. *cereus* F837/76 (bcf_08380 = BCF_RS08250) without a putative N-terminal signal peptide for secretion was cloned into the vector pASK-IBA5+ (IBA Lifesciences, Göttingen, Germany) using the primers flaA-fw-KpnI (ATA TGG TAC CGC GCA TCA ATA GTG CTG) and flaA-rev-NcoI (ATA TCC ATG GTT ATT GTA ATA ATT TAC TTA CC). For determination of the binding site of mAb 1A11, five overlapping *flaA* fragments were cloned analogously, *flaA1* (ATA TGG TAC CGC GCA TCA ATA GTG CTG and ATA TCC ATG GTT AAC CAT CTA AAG CGT C), *flaA2* (ATA TGG TAC CGG AAT ACC AAC AGC TAA TTA C and ATA TCC ATG GTT ATG CAT CTA TCA TTG TAT C), *flaA3* (ATA TGG TAC CGA TTG CAG GAA ACC GAG and ATA TCC ATG GTT ATT GTA ATA ATT TAC TTA CC), *flaA4* (ATA TGG TAC CGC AAG AAA GCG GGT TAA ATG TC and ATA TCC ATG GTT AAA TTG TAC TAG CTT TTG ATT TAT C) and *flaA5* (ATA TGG TAC CGG ACA TCG AAA CTA AAG CAG and ATA TCC ATG GTT ACA TTT GTG GAG TTT GGT TTG). Protein overexpression and purification via the N-terminal strep-tag was conducted as described before [38].

### Generation of rabbit antisera and mouse monoclonal antibodies (mAbs)

Generation of rabbit antisera was conducted by an external provider. Animal husbandry and all experiments for antibody generation were in accordance with the German Law for Protection of Animals. All procedures and protocols were approved by the ethics committee of the Government of Upper Bavaria. Female ZIKA hybrid rabbits, 12 weeks old, were immunized with purified, recombinant flagellin preparations using standard procedures [40]. Primary injections (s.c.) were with 100 μg protein emulsified in Freund’s complete adjuvant. Rabbits were boostered at four weeks intervals (50 μg protein in Freund’s incomplete adjuvant, s.c.) and blood samples were taken 10 d after the booster injections by puncture of the lateral ear vein. After the final booster, animals were bled by cardiocentesis under pentobarbital anaesthesia.

A group of five female hybrid mice [BALB/c x (NZW x NZB)] was immunized several times with purified recombinant flagellin protein (20 μg/animal) emulsified in Sigma adjuvant (wk 0 and 5) or incomplete Freund’s adjuvant (subsequent immunizations). The mice exhibiting the best immune response (i.e. high antibody titres, high affinity) were chosen for the production of mAbs. Three days after injection of 30 μg rFla, dissolved in PBS, spleen cells were fused with myeloma cells (P3-X63-Ag8.653). Ten days after fusion, clones were assayed for antibody production by indirect EIA using rFla (0.25 μg/ml) as coating antigen. Further establishment of reactive hybridomas, mass production and antibody purification were performed as previously described [41, 42]. Mice were housed on a 14-h light, 10-h dark cycle with free access to water and standard chow diet. All studies were carried out in strict accordance with directive 2010/63/EU on the protection of animals used for scientific purposes. Mice used as spleen donors were sacrificed by cervical dislocation.

### Labelling with HRP (horseradish peroxidase)

For use as detection antibodies in the sandwich EIAs, the rabbit serum was coupled to activated peroxidase (HRP) according to the instructions of the manufacturer (Roche; Merck, Darmstadt, Germany). The resulting conjugate was stabilized with 1% BSA and StabilZyme® HRP Conjugate Stabilizer (SurModics, Eden Prairie, USA) and conserved with 0.01% Thimerosal.

### SDS PAGE and Western blotting

SDS-PAGE was performed on Novex^TM^ WedgeWell^TM^ 8–16% Tris-Glycine gels (Invitrogen; Thermo Fisher Scientific, Waltham, USA) for 90 min at 125 V. After electrophoresis, proteins were blotted to a PVDF-P membrane (Millipore; Merck, Darmstadt, Germany), which was blocked in 3% casein-PBS and incubated for 1 h at room temperature with 2 μg/ml mAb 1A11 or with the rabbit antiserum (1:1000). The membrane was washed three times in PBS with 0.1% Tween 20 and incubated overnight with rabbit anti-mouse- or goat anti-rabbit-HRP (horseradish peroxidase) conjugate (Dako; Merck, Darmstadt, Germany) diluted 1:2000 in 1% casein-PBS. After three further washing steps in PBS with 0.1% Tween 20 and two in PBS, Super Signal Western Femto (Pierce; Thermo Fisher Scientific, Waltham, MA, USA) was applied and chemiluminescence signals were detected on a UVP ChemStudio imager (Analytik Jena, Jena, Germany).

### Enzyme Immunoassays (EIAs)

Indirect and sandwich enzyme immunoassays for the detection of flagellin were established. In the indirect assay, recombinant FlaA protein as well as prepared culture supernatants (see above) were applied to the microtitre plate in bicarbonate buffer (1.59 g/l Na_2_CO_3_, 2.93 g/l NaHCO_3_) as serial dilution (100μl/well, start amounts 1 μg/ml and 1:60, respectively). After overnight incubation at room temperature, 30 min blocking with 150 μl/well 3% sodium-caseinate-PBS and 3x washing (wash buffer: 146 mM NaCl, 0.025% Tween 20), mAb 1A11 or the rabbit serum was applied in PBS (100μl/well, 2 μg/ml and 1:200 dilution, respectively). The plates were incubated for 1 h at room temperature on an orbital platform shaker. After four washing steps, rabbit anti-mouse- or goat anti-rabbit-HRP conjugate (Dako; Merck, Darmstadt, Germany) was added as secondary antibody 1:2000 in 1% sodium-caseinate-PBS. After further incubation for 1 h on the orbital platform shaker, the microtitre plate was washed five times and 100μl/well 5% TMB (tetramethylbenzidin)-solution in citrate buffer were applied. The reaction was stopped after 20 min by addition of 1 M sulfuric acid (100μl/well) and absorbance at 450 nm was measured immediately in a Tecan infinite F50 photometer using Magellan software (Tecan Group Ltd., Männedorf, Switzerland). Titres are defined as the reciprocal of the highest dilutions resulting in an absorbance value of ≥1.0.

In the sandwich EIA, the microtitre plate was coated with mAb 1A11 (10 μg/ml in PBS). Samples were applied in PBS with 0.5% Tween 20 as serial dilution (start amount 0.3 μg/ml FlaA proteins or 1:2 dilution of bacterial culture supernatants). For detection, HRP-labelled rabbit antiserum was used in 1:500 dilution in 1% sodium-caseinate-PBS. Incubation, blocking, washing and detection were carried out as described above for the indirect assay.

### Immunofluorescence

*B*. *cereus* cell pellets were washed twice in 1 ml PBS with interjacent centrifugation steps (3 min, 8.000 g, room temperature) and subsequently resuspended in 500 μl PBS with 1% Tween 20. Rabbit antiserum was added in 1:50 dilution before the mixtures were incubated for 45 min at room temperature on an overhead shaker with moderate agitation (2 rpm). After samples were washed twice in 500 μl PBS, the cell pellets were resuspended in 500 μl PBS containing 2 μg/ml Alexa 488 goat anti rabbit IgG (life technologies, Carlsbad, USA). Samples were incubated in the dark for 45 min at 4°C on an overhead shaker with moderate agitation (2 rpm). After that, they were again washed twice and resuspended in 250 μl PBS. Five μl samples were examined at 488 nm in a Biozero BZ8000 microscope (Keyence, Neu-Isenburg, Germany).

### Motility and growth tests

Motility of different *B*. *cereus* strains was assessed by investigating swimming behaviour in CGY medium supplemented with 0.25% agar. If appropriate, 10 and 20 μg/ml mAb 1A11 as well as equal volumes of the rabbit antiserum were added. One μl of a CGY overnight culture each was injected at the centre of the 53 mm plate. After 24 h of incubation at 37°C, swimming diameters of three replicates were measured.

Growth experiments under addition of flagellin-specific antibodies were carried out in triplicates in 96-well microtitre plates (200 μl/well) in CGY medium for eight h. Plates were incubated at 37°C on an orbital platform shaker. Medium was inoculated to OD_600_ = 0.2 and OD_600_ was measured every hour using a Tecan infinite F50 photometer (Tecan Group Ltd., Männedorf, Switzerland).

### Electron microscopy

Sample preparation for scanning electron microscopy (SEM) was carried out as described previously [43] and included the following steps: application to glass slides, rapid freezing with liquid nitrogen, a following chemical fixation with 2.5% glutaraldehyde, post-fixation with 1% osmium tetroxide, dehydration with a graded acetone series and critical point drying. After application of a 3 nm layer of platinum via sputter-coating, the samples were investigated with a Auriga high-resolution SEM (Carl Zeiss Microscopy, Jena, Germany) operated at 2 kV.

### RNA preparation, reverse transcription and assessment of flagellin gene expression

Total RNA preparation and on-column DNase digestion were performed using RNeasy Mini Kit and RNase-Free DNase Set (QIAGEN, Hilden, Germany) according to the instructions of the manufacturer. RNA quality was confirmed via spectrophotometer and agarose gel electrophoresis. RNA purity was tested via PCR for a 241 bp 16S rRNA fragment using the primers 16S_fw (GGA GGA AGG TGG GGA TGA CG) and 16S_rev (ATG GTG TGA CGG GCG GTG TG). Double-stranded cDNA was synthesized from 100 ng RNA templates using the ProtoScript first-strand cDNA synthesis kit (New England Biolabs, Frankfurt, Germany) with random primer mix according to the recommendations of the manufacturer. The cDNA was subsequently applied in a PCR amplifying a 159 bp fragment of the flagellin gene using the primers fla-5’-fw (ATG AGA ATT AAT ACA AAC ATT AAC AG) and fla-5’-rev (ACG CAT ACG AGT TGC GAT TGC). The fragment represents the highly conserved 5’ part of the gene (compare also amino acid alignment in point C in S2 Fig).

### Sequencing of flagellin promoters

Using the primers flaP-up (CTGAATTTGTCCTTTCTTATATG) and flaP-down (GGTTTTGGCGCATGTACTC), a 371 bp DNA fragment upstream of the flagellin gene was amplified via PCR. This included the flagellin promoter sequence (obtained from bcf_08380) as well as 60 additional bp up and downstream. Chromosomal DNA of the *B*. *cereus* strains F837/76, F837/76_2 and IP5832 was used as template. The DNA fragments were sequenced via the Tube Seq service of Eurofins Genomics (Ebersberg, Germany).

### *In silico* analyses

Statistical data analyses were performed using the column statistics program of GraphPad Prism Version 5.04 for Windows, GraphPad Software, San Diego California USA, www.graphpad.com. For EIA and motility results, XY data were entered and XY analyses were performed. For correlation tests, no Gaussian distribution was assumed and nonparametric Spearman correlation with 95% confidence interval was computed. For the results of EIAs with rFla fragments, non-linear regression was performed using the one site—specific binding model of GraphPad Prism. The flagellin genes of the sequenced *B*. *cereus* strains [36, 44] were determined using mauve version 20150226 [45] and strain F837/76 (GenBank: CP003187.1) as reference. DNA and protein sequences were aligned using Clustal Omega [46]. Structural models of *B*. *cereus* F837/76 flagellin were generated using SWISS-MODEL [47].

## Results

### Generation of polyclonal and monoclonal antibodies (mAbs) against flagellin

The flagellin gene from *B*. *cereus* strain F837/76 (bfc_08380) was cloned into the expression vector pASK-IBA5+. The corresponding 27.2 kDa protein was overexpressed in *E*. *coli* and purified via its N-terminal strep-tag. First, two rabbits were immunized, resulting in the two polyclonal antisera #5320 and #5321. Second, the protein was used for immunization and two booster injections of mice. After cell fusion, a hybridoma cell line secreting the highly specific mAb 1A11 (IgG1, κ) could be identified. Subsequently, this mAb was mass-produced and purified according to established procedures [41, 42]. Using this mAb, recombinant flagellin protein (rFla) could be detected in concentrations of < 2 ng/ml under the conditions of an indirect enzyme immunoassay (EIA).

### Detection of the rFla and flagellin from bacterial cultures

**Fig 1:**
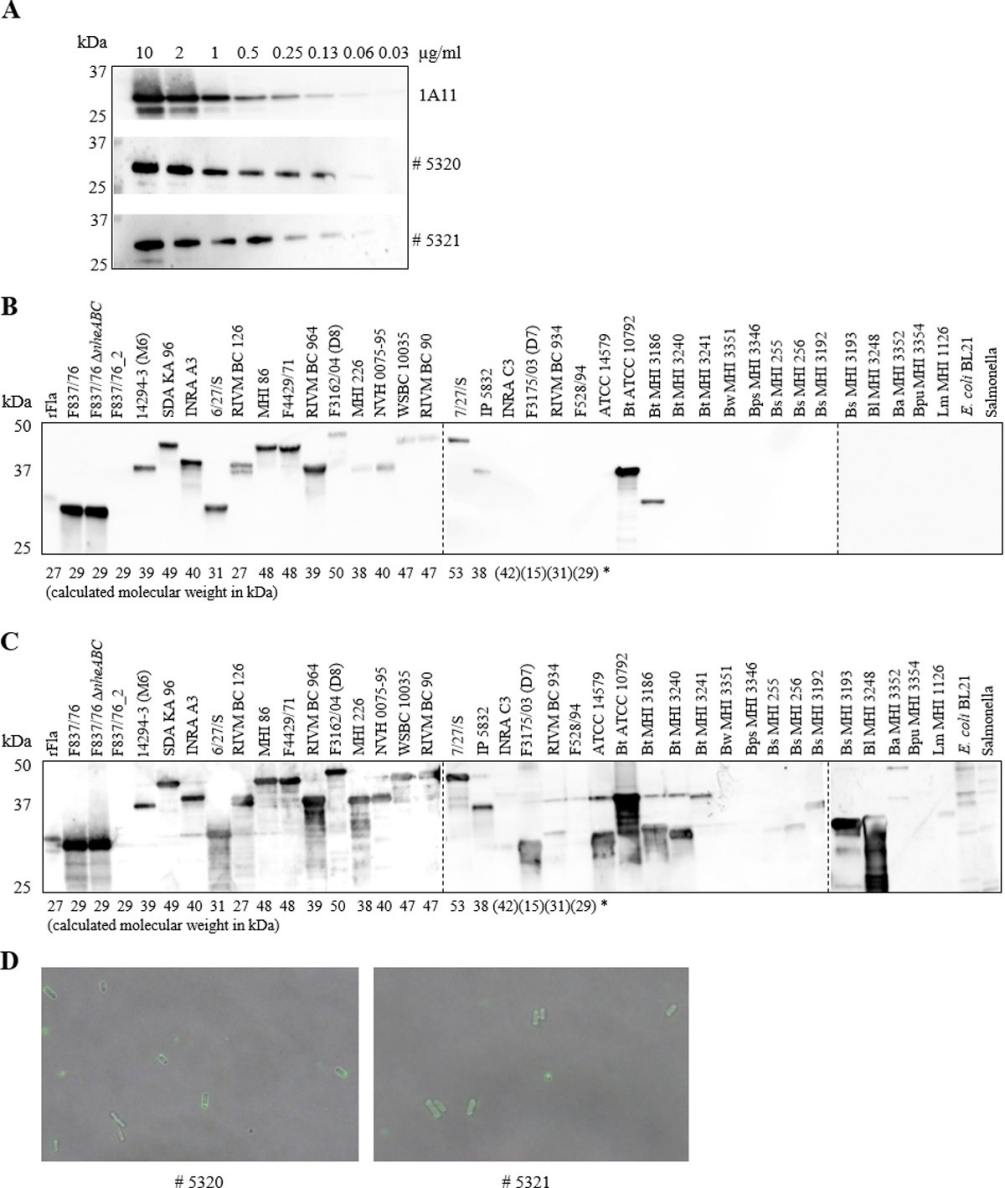
Detection of flagellin by the generated rabbit antisera and the monoclonal mouse antibody 1A11. (**A**) Recombinant flagellin in Western blots. Rabbit antisera were applied 1:1000, mAb 1A11 as 2 μg/ml. (**B**) mAb 1A11-based detection of flagellin in the supernatant of selected bacterial cultures. 20 μl of culture supernatants of 23 *B*. *cereus* strains as well as different controls were applied. Bt: *B*. *thuringiensis*, Bw: *B*. *weihenstephanensis*, Bps: *B*. *pseudomycoides*, Bs: *B*. *subtilis*, Bl: *B*. *licheniformis*, Ba: *B*. *amyloliquefaciens*, Bpu: *B pumilus*, Lm: *L*. *monocytogenes*. * three flagellin proteins exist with molecular weights of 31, 32 and 34 kDa [9]. (**C**) Rabbit serum # 5321-based detection of flagellin in the supernatant of selected bacterial cultures. Application as in B. (**D**) Application of the rabbit sera in immunofluorescence. Strain WSBC 10035 is shown as an example.

The two polyclonal antisera and mAb 1A11 detected the rFla protein in Western blots up to a concentration of approx. 0.06 μg/ml (Fig 1A). When bacterial samples were used, mAb 1A11 appeared to be highly specific towards *B*. *cereus* flagellin, with the exception of two (closely related) *B*. *thuringiensis* strains (Fig 1B). The rabbit antisera, however, showed a broader reactivity and bound also to some *B*. *thuringiensis* and *B*. *subtilis* flagellins (see Fig 1C, serum # 5321 as an example). Nevertheless, the sera could be used for immunofluorescence-based detection of *B*. *cereus*, which is shown for strain WSBC 10035 as an example (Fig 1D). Notably, there was a high variability in the molecular weights of the detected flagellins, which, to a large extent, correlate to the molecular weights calculated from available flagellin protein sequences (compare point C in S2 Fig). It is particularly interesting that the N- and C-terminal parts seem to be highly conserved, while strain-specific variations appear especially in the middle section of the proteins.

### Establishment of enzyme immunoassays for flagellin detection

Initially, indirect EIAs for the detection of flagellin from bacterial samples were developed based on using recombinant flagellin protein (see above). For testing natural culture supernatants, the *B*. *cereus* strains were grown for 6 h in CGY full medium at 37°C, as earlier studies showed uniformly increased swimming motility at this temperature [26]. Thus, under these growth conditions, the EIA yield (flagellin titres) was maximized. It can further be assumed that the applied growth conditions are not responsible for strain-specific variations of flagellin titres in the EIAs.

**Fig 2:**
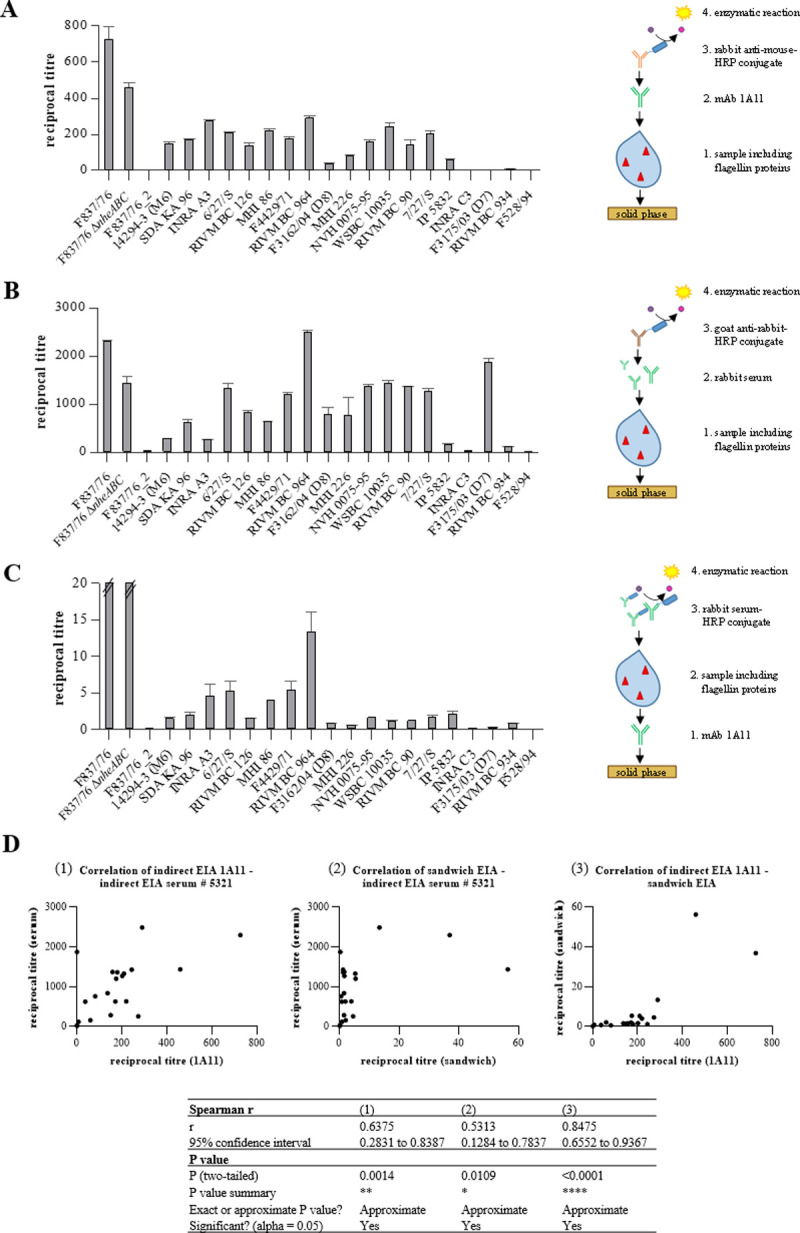
Application of the generated antibodies in EIAs. Shown are the results (reciprocal titres) obtained for 22 *B*. *cereus* strains by using (**A**) Indirect EIAs with mAb 1A11, (**B**) Indirect EIAs with rabbit serum # 5321, and (**C**) Sandwich EIAs. Results indicate means and standard deviations of two biological with three technical replicates for each strain. (**D**). For correlation tests of EIA results, nonparametric Spearman correlation with 95% confidence interval was computed using GraphPad Prism. These calculations were performed after first computing the mean of side-by-side replicates, and then analysing those means.

In the indirect assays with mAb 1A11, flagellin proteins of 22 *B*. *cereus* strains were detected with varying strength. Reciprocal titres reached from 0 (F528/94) to 726.1 ± 118.7 (F837/76) (Fig 2A). A similar pattern was observed for the indirect EIAs with rabbit antiserum # 5321, where reciprocal titres ranged from 19.9 ± 0.7 (F528/94) to 2489.7 ± 65.9 (RIVM BC 964) (Fig 2B). As observed before in Western blots (compare Fig 1B and 1C), mAb 1A11 showed almost no cross-reactivity with other bacteria outside of the *B*. *cereus* group, while distinct reciprocal titres were obtained with the rabbit serum (points A and B in S3 Fig). Subsequently, the two approaches were combined in a highly specific sandwich EIA where mAb 1A11 served as primary antibody, while the higher affine HRP-labelled rabbit serum #5321 was used for detection. Applying this EIA for analysis of a broad range of Bacilli, almost no cross-reactivity was observed (point C in S3 Fig), and the distinct pattern in *B*. *cereus* flagellin detection was again obvious with reciprocal titres from 0 (F837/76_2) to 56.3 ± 15.2 (F837/76 Δ*nheABC*) (Fig 2C). Moreover, Spearman correlation tests showed that the data obtained from both indirect assays significantly correlated (r = 0.6375), as did the indirect assay (serum # 5321) with the sandwich EIA (r = 0.5246). Best correlation was calculated for the indirect assay (1A11) and the sandwich EIA (r = 0.8475) (Fig 2D).

### Determination of the binding site of mAb 1A11

**Fig 3:**
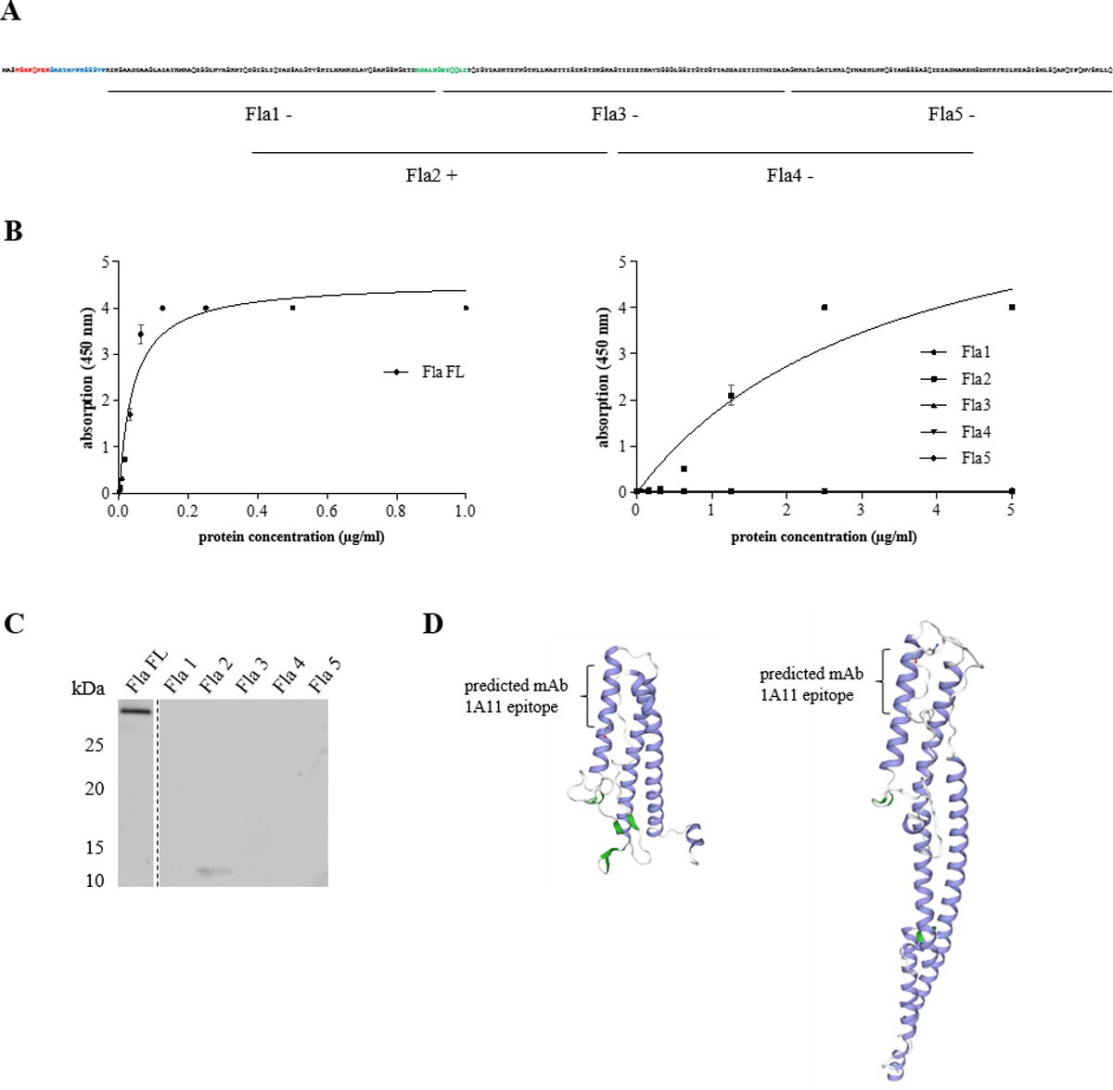
Determination of the binding site of mAb 1A11. (**A**) Schematic overview of the five truncated rFla protein fragments (Fla1-Fla5). Red: strep-tag, blue: linker, green: mAb 1A11 epitope. (**B**) Results of indirect EIAs. Full length (FL) flagellin was used as serial dilution from 1 μg/ml, the truncated fragments from 5 μg/ml. Results indicate means and standard deviations of three replicates for each protein. Non-linear regression was performed using the one site—specific binding model of GraphPad Prism. (**C**) Results of Western blotting. Full length (FL) recombinant flagellin was applied in a concentration of 0.25 μg/ml, the truncated fragments Fla1-Fla5 as 20 μg/ml. (**D**) Models of *B*. *cereus* F837/76 flagellin (bcf_08380). Protein structures were generated using SWISS-MODEL [47]. Left: Crystal structure of *Bacillus cereus* flagellin (5z7q.1.A) was used as template, which showed approx. 60% coverage and at the same time the highest sequence identity of all available templates (50.3%). Right: Cryo-EM structure of *B*. *subtilis* flagellar filaments N226Y (5wjt.1.A) was used as template, which showed 100% coverage and at the same time 45.52% sequence identity.

The high specificity of the mAb-based tools prompted us to identify the flagellin epitope reactive with mAb 1A11. For this, five truncated, overlapping fragments of F837/76 flagellin were constructed, overexpressed and purified analogously to the full-length (FL) protein (see Fig 3A and point D in S2 Fig). In indirect EIAs as well as in Western blots, only the fragment rFla2 could be detected (Fig 3B and 3C), which suggests that the epitope is located between amino acids 97 and 108 of the recombinant protein and between amino acids 107 and 118 of the wild type flagellin (RDALDGEYQQLI, see also point D in S2 Fig). As no protein structure of *B*. *cereus* F837/76 flagellin exists, models were created using two different templates with either highest available sequence identity or coverage and identity. The position of the predicted mAb 1A11 epitope is marked (Fig 1D). However, it has to be considered that the signals received from the fragment rFla2 (in Western blots as well as EIAs) were rather weak compared to the full-length flagellin, while much higher protein concentrations were loaded. Furthermore, mAb 1A11 recognized a great variety of strains with low sequence homology in that region (compare Figs 1B, 2, and point C in S2 Fig). These findings rather point to a conformational instead of a linear epitope.

### Strain-specific swimming motility

**Fig 4:**
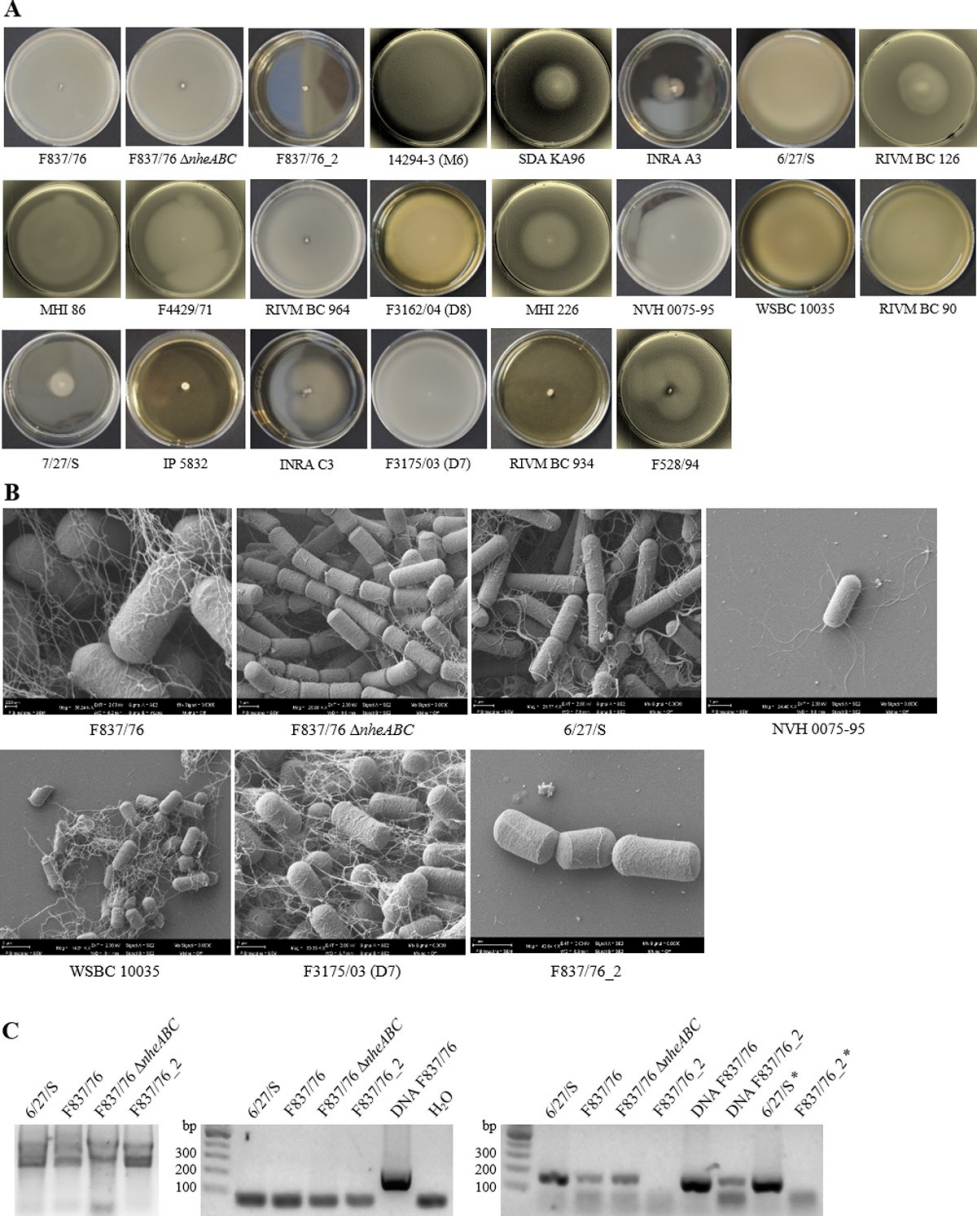
Flagella-driven motility of selected *B*. *cereus* strains. (**A**) Swimming motility on CGY plates supplemented with 0.25% agar after 24 h incubation at 37°C. Each strain was tested at least in triplicates. One representative image per strain is shown. Images were obtained from this study or from [26]. (**B**) Scanning electron microscopy of seven selected strains. (**C**) Flagellin gene expression of four strains, which were grown for 3 h in CGY full medium. Gel left: total RNA on 1% agarose. Gel middle: Control PCR of a 241 bp fragment of the 16S rRNA gene on 1% agarose. No residual DNA was detected in the samples. Gel right: After reverse transcription of the RNA samples, a 159 bp, N-terminal fragment of the flagellin gene was amplified via PCR and is shown on 1% agarose. *: cDNA of strains 6/27/S and F837/76_2 previously grown under simulated intestinal conditions [37].

In an earlier study, we observed highly strain-specific swimming and swarming motility of a selected set of enteropathogenic and apathogenic *B*. *cereus* isolates [26]. This was extended by newly investigated strains in this study. Fig 4A shows the strain-specific swimming motility in CGY with 0.25% agar after 24 h incubation at 37°C. One representative image is depicted for each strain. Generally, swimming correlated well with the detection of flagella, which were visualized for seven selected strains via scanning electron microscopy (SEM). Interestingly, a variant of strain F837/76 (F837/76_2) seemed to have lost its flagella and thus, its swimming ability (Fig 4A and 4B). To find the reason for the dysfunctional flagellin production, total RNA was prepared and reverse transcribed into cDNA, which was subsequently used as template for amplification of a 159 bp, N-terminal fragment of the flagellin gene (Fig 4C). The fragment was amplified when chromosomal DNA of F837/76_2 was used, indicating that the flagellin gene is still present in this strain. Compared to the highly motile and flagellated strains 6/27/S, F837/76 and F837/76Δ*nheABC*, no signal could be detected applying cDNA of F837/76_2. This was observed for growth in CGY full medium as well as under “simulated intestinal conditions” (RPMI 1640 medium pre-incubated with CaCo-2 cells, 37°C, 7% CO_2_ atmosphere; [37]). Interestingly, when we retrospectively amplified and sequenced the flagellin promoter regions of strains F837/76 and F837/76_2, they proved to be identical (compare point E in S2 Fig). These observations suggest that this particular strain no longer expresses the flagellin gene for yet unknown reasons.

**Fig 5:**
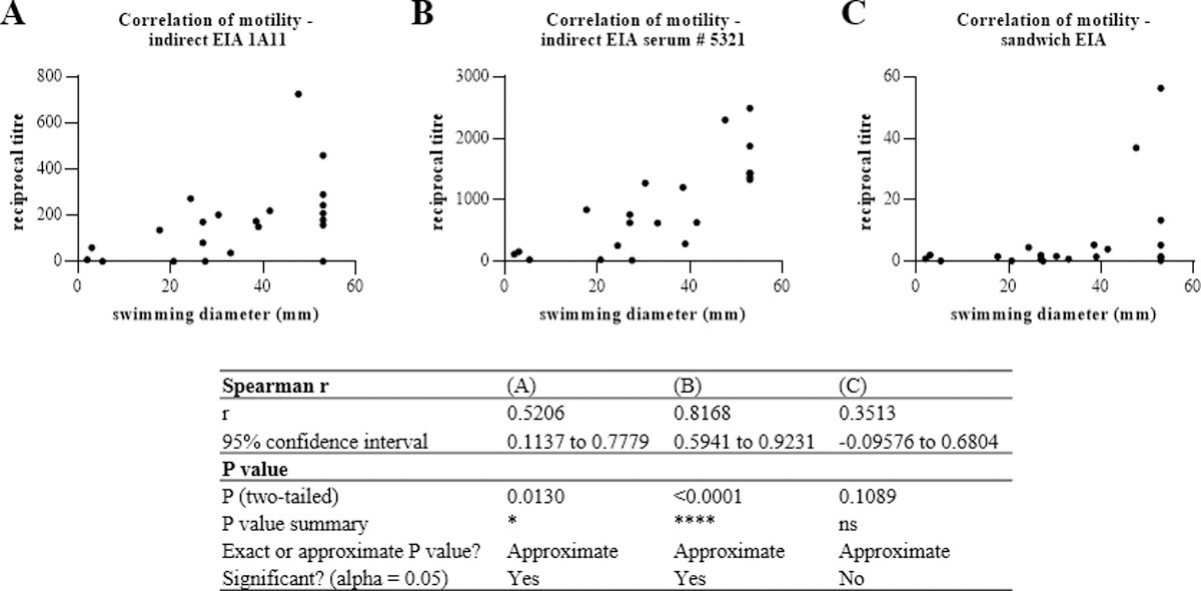
Correlation between flagellin titres in EIAs and swimming motility of 22 tested *B*. *cereus* strains. Swimming diameters (in triplicates), determined in an earlier [26] and in this study, were compared to the EIA data (in triplicates) obtained in this study using nonparametric Spearman correlation with 95% confidence interval in GraphPad Prism. These calculations were performed after first computing the mean of side-by-side replicates, and then analyzing those means.

Furthermore, swimming motility of most tested strains was in agreement with the detection of flagellin with the EIAs developed in this study (see above and Table 1 for data overview). Exceptions were strains INRA C3, F3175/03 (D7) and F528/94. Swimming diameters ([26] and this study) generally correlated with the indirect EIAs (1A11: r = 0.5206 and rabbit serum # 5321: r = 0.8168). A slight, but insignificant correlation was also calculated for the sandwich EIA (r = 0.3513) (see also Fig 5).

**Table 1:** Comparative overview of the data of 22 *B*. *cereus* strains obtained in this study.

Strain	Flagellin (amino acids)	Flagellin (kDa)	Western blot mAb 1A11	Western blot serum # 5321	Indirect EIA mAb 1A11	Indirect EIA serum # 5321	Sandwich EIA	Swimming*
F837/76	268	28.74	+	+	++	++	++	++
F837/76 Δ*nheABC*	268	28.74	+	+	++	+	++	++
F837/76_2	268	28.74	-	(-)	-	(-)	(-)	(-)
14294–3 (M6)	346	39.00	+	+	+	+	+	+
SDA KA96	457	48.59	+	+	+	+	+	+
INRA A3	371	40.42	+	+	+	+	+	+
6/27/S	287	31.02	+	+	+	+	+	++
RIVM BC 126	249	26.71	+	+	+	+	+	+
MHI86	446	47.56	+	+	+	+	+	+
F4429/71	446	47.56	+	+	+	+	+	+
RIVM BC 964	364	39.26	+	+	+	++	++	++
F3162/04 (D8)	465	49.85	+	+	+	+	+	+
MHI226	363	38.38	+	+	+	+	+	+
NVH 0075–95	373	39.96	+	+	+	+	+	++
WSBC 10035	445	47.50	+	+	+	+	+	++
RIVM BC 90	445	47.50	+	+	+	+	+	++
7/27/S	494	52.57	+	+	+	+	+	+
IP 5832	359	38.18	+	+	+	+	+	(+)
INRA C3	393	42.43	-	+	-	(-)	(+)	+
F3175/03 (D7)	142	15.23	-	+	-	++	(+)	++
RIVM BC 934	287	31.02	-	+	(-)	(+)	+	-
F528/94	272	29.27	-	(-)	-	-	-	+

*: according to [26] and this study. ++: very strong signal, +: strong signal, (+): weak signal, (-): very weak signal, -: no signal.

### Influence of the generated antibodies on motility and growth

**Fig 6:**
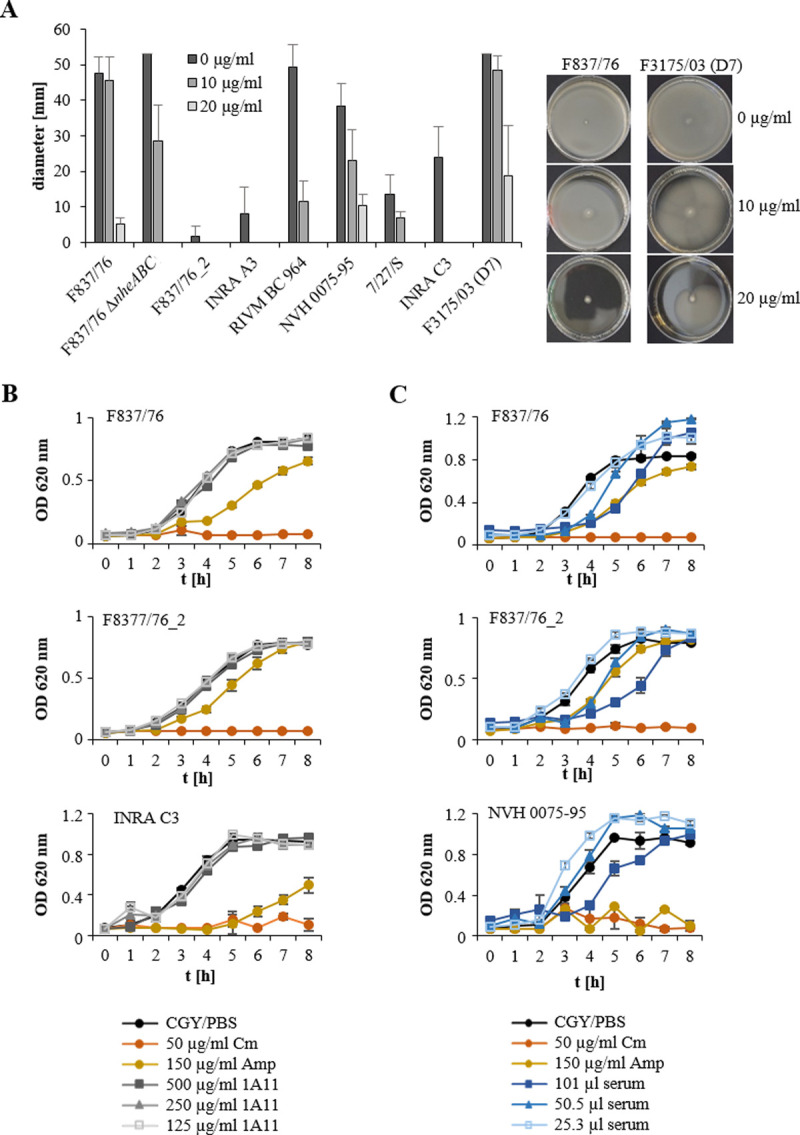
Motility and growth of different *B*. *cereus* strains under addition of anti-flagellin antibodies. (**A**) Swimming motility of nine *B*. *cereus* strains on CGY plates supplemented with 0.25% agar after 24 incubation at 37°C. mAb 1A11 was added to the agar in concentrations of 10 and 20 μg/ml, respectively. (**B**) Growth of three selected *B*. *cereus* strains in CGY medium. Medium was mixed 1:1 with PBS containing the appropriate concentration of mAb 1A11 or antibiotics as control. (**C**) Growth of three selected *B*. *cereus* strains in CGY medium. Medium was mixed 1:1 with PBS containing the same volume (in μl) of rabbit serum # 5321 as used for mAb 1A11. Results of swimming and growth assays are depicted as means and standard deviations of two independent runs with three technical replicates each.

After getting first insights into the highly strain-specific swimming motility of the tested *B*. *cereus* strains, the newly generated, flagellin-specific antibodies were added to the CGY soft-agar to determine their possible influence on swimming motility. mAb 1A11 proved to hinder bacterial swimming. This inhibition was concentration-dependent, but also strain-specific differences were observed once more. While 10 μg/ml mAb 1A11 completely hindered motility of strains INRA A3 and INRA C3, strains F3175/03 (D7), NVH 0075–95 or F837/76 were still partly motile even after applying 20 μg mAb per ml (Fig 6A). Interestingly, when growth of the nine selected strains was tested in CGY medium under addition of the antibodies, the opposite was demonstrated. mAb 1A11 had no significant influence on growth (Fig 6B, shown for three strains). Lower amounts of serum #5321 seemed to have no effect or slightly enhance growth, while higher amounts of the serum delayed growth (Fig 6C, shown for three strains). In contrast to motility, strains F837/76 and F837/76_2 responded equally to the different growth conditions.

## Discussion

In the present study, antibody-based tools for specific investigation of *B*. *cereus* flagella and especially flagellin were developed. Using polyclonal rabbit antisera as well as mouse mAb 1A11, indirect as well as sandwich EIAs for flagellin detection were established. As a first result, for the presence of flagella, particularly flagellin titres in indirect EIAs, a significant correlation with swimming motility of 22 *B*. *cereus* strains could be determined. Only exceptions were strains INRA C3, F3175/03 (D7) and F528/94. INRA C3 showed moderate swimming at 30 and medium swimming at 37°C [26]. In this strain, flagellin could be moderately detected with the rabbit serum, but not with mAb 1A11 (this study). F3175/03 (D7) showed excellent swimming at 30 and 37°C [26], while flagellin could be clearly detected with the rabbit serum, but not with mAb 1A11 (this study). For F528/94 on the contrary, flagellin could neither be detected with the rabbit serum nor with mAb 1A11 (this study), and the strain showed medium swimming motility at 37, but no swimming at all at 30°C [26] (refer also to Table 1 for data comparison). Comparing the flagellin protein sequences of 20 strains, it became obvious that in the N-terminal, rather conserved part, strain F3175/03 (D7) bears an exceptionally high number of amino acids substitutions compared to other strains (point C in S2 Fig). Furthermore, this sequence results in a flagellin protein of only 15.23 kDa, which is also not in accordance with data from Western blots obtained in this study (compare Fig 1B and 1C). Nevertheless, for most other available sequenced strains, calculated molecular weights and kDa obtained in Western blots matched well (see Fig 1B and 1C and point C in S2 Fig). The only further exception was strain RIVM BC 126, for which a molecular weight of 26.71 kDa was calculated. Instead of this, two distinct bands at >37 kDa were detected. Further, slight variations might be the result of flagellin glycosylation [48, 49]. Of special interest is the great strain-specific variability of molecular weights reaching from 29 to 53 kDa. Generally, when the draft genome sequenced [36, 44] were searched for homologues of bcf_08380, only one corresponding gene was found. This is in accordance with earlier studies describing one or a maximum of two (one active, one inactive) flagellin encoding genes for most tested *B*. *cereus* strains. Type strain ATCC 14579 seems to be atypical with four flagellin gene homologues, of which three encode flagellin proteins [9]. Differences in swimming motility and in flagellin protein expression might also result from variations in the promoter regions of the corresponding genes. Generally, this genetic element appeared rather conserved in the 21 *B*. *cereus* strains compared in this study (refer to point E in S2 Fig). The -35 region for instance was completely conserved, while in the -10 region one bp substitution was detected in strain NVH 0075–95. +1 is missing in strain RIVM BC 126, which also misses further bp, as well as strain F3175/03 (D7). Interestingly, although no flagella (or flagellin gene expression) could be detected in the variant F837/76_2, the promoter sequence was identical to that of F837/76.

Detection of bacterial flagella via specific antibodies has a long tradition. In the 1960s and 70s, classification schemes for both *B*. *thuringiensis* and *B*. *cereus* have been developed, based on the interaction of polyclonal antisera with the flagellar (H) antigen, first in agglutination tests, later via EIAs. By 1999, 69 *B*. *thuringiensis* serotypes and 13 sub-antigenic groups existed [50]. For *B*. *cereus*, first flagellar serotypes H1-H23 were defined [51, 52], before further serotypes T1-T12 were added, while a great percentage of the tested isolates remained untypable [53]. Efforts were undertaken to connect serotyping with the epidemiology of *B*. *cereus* food poisoning and to use this for differentiation between emetic and enteropathogenic isolates. While the majority of food poisoning strains could be assigned to serotype H1 or H8 and approx. 90% of the emetic isolates to H1 [52, 54], a multitude of food isolates was untypable [52, 53]. It has further been shown that an increasing number of *B*. *cereus* strains cross-reacts with the generated *B*. *thuringiensis* H antisera. Thus, the two species are not necessarily distinguishable by flagellar serotyping [50, 55, 56]. This is not surprising considering their close genetic relationship [57, 58]. In the present study, three previously serotyped strains were applied as controls. *B*. *cereus* ATCC 14579, which had been classified as “untypable”, was detected by the rabbit serum #5321 at >30 kDa, which matches prior observations [9]. No signal was detected when mAb 1A11 was used (compare Fig 1B and 1C). Similarly, strain MHI 3240, also known as *B*. *thuringiensis* ssp. *kurstaki* HD-1, was detected by the rabbit serum at approx. 30 kDa, but not by mAb 1A11 (Fig 1B and 1C). This strain was designated before as serotype H3 [55] or H3a, 3b, 3c [50]. In contrast to that, *B*. *thuringiensis* ATCC 10792, Berliner, assigned to serotype H1 [50, 55], was detected by both, serum #5321 and mAb 1A11 at approx. 37 kDa (Fig 1B and 1C). Thus, the rabbit serum #5321 cross-reacts with different specified and unspecified serotypes, as well as with further species such as *B*. *subtilis* or *B*. *licheniformis* (compare Fig 1C and point B in S3 Fig). On the contrary, mAb 1A11 seems to bind rather specifically to isolates of serotype H1. This might be one reason why strains INRA C3, F3175/03 (D7), RIVM BC 934 and F528/94 were not detected in Western blots (Fig 1B) and only barely in EIAs (Fig 2). These strains were isolated from pasteurized carrots, human faeces, lettuce and a rice dish in connection with food poisoning, respectively [36]. The other 16 strains recognized by mAb 1A11 were of different origin including foods (n = 8), human faeces after food poisoning outbreaks (n = 5), one postoperative infection, one probiotic, and one dish in connection with an outbreak [36]. Thus, in contrast to previous assumptions (see above), the present study was not able to differentiate between food and food poisoning isolates by means of flagellar detection.

Nevertheless, highly strain-specific variations, not only in toxin production or the ability to provoke the diarrhoeal syndrome [26], but also in motility, flagellar composition, and flagellin protein sequences became obvious. With the exception of strain F3175/03 (D7), the N- and C- terminal parts of the flagellin proteins are rather conserved, while most variation appear in the middle section (compare point C in S2 Fig). This has also been shown in an earlier study, where the first 111 and the last 66 amino acids of flagellin of 106 selected *Bacillus* strains were conserved, while the central region was highly variable. It was also stated that H-serotypes do not necessarily correlate with flagellin amino acid sequences [56].

In the present study, also the binding site of mAb 1A11 could be identified, which lies in the rather conserved, N-terminal region. Nevertheless, strain-specific amino acid variations appear which might be another explanation why some strains were not recognized by the antibody including *B*. *cereus* type strain ATCC 14579 (compare points B and C in S2 Fig). Next to flagellar detection, another special feature of mAb 1A11 is that it can be utilized for inhibition of flagella-driven swimming motility (compare Fig 6A). By this, the ability of the pathogen to actively move towards and to colonize the site of infection can be seriously disrupted, leading to generally decreased pathogenicity. Extensive research is performed to generate vaccines and novel antimicrobial strategies based on the impairment of flagellar functions, such as motility and adhesion, for pathogenic bacteria such as *Campylobacter*, *Helicobacter* or *Pseudomonas aeruginosa* [59, 60]. The development of further anti-infective strategies by counteracting bacterial flagella-driven swarming behaviour is also focus of attention. Among other things, modulators can be fatty acids, secondary plant metabolites, enzymes or phages, while the majority of these molecules seems to interfere with flagellar gene expression [17].

Altogether, in the present study a powerful tool for the specific detection and impairment of *B*. *cereus* flagella was developed.

## Supporting information

S1 Fig Uncropped and unadjusted images of blots and gels. X indicates parts of the images not used for the manuscript figures. Chemiluminescence signals were detected on a UVP ChemStudio imager (Analytik Jena, Jena, Germany) after 20–120 s exposure. Samples on 1% agarose gels were made visible on a UV table and photographed with a Huawei Psmart cell phone.
(PDF)

S2 Fig Sequences and multiple sequence alignments (CLUSTAL O 1.2.4) of *B*. *cereus* flagellin. **A.** Comparison of bcf_08380 (strain F837/76) and the three verified flagellin-encoding genes of strain ATCC 14579. **B.** Alignment of the corresponding protein sequences. The epitope most likely recognized by mAb 1A11 is marked in green. **C.** Alignment of the bcf_08380 gene product and its homologues of 19 enteropathogenic and apathogenic B. cereus strains. bcf_08380 is highlighted in bold. The mAb 1A11 epitope is shown in green. Strains INRA C3 and F3175-03_(D7) are underlined. Amino acid substitutions in the N-terminal region are highlighted (red: only strain F3175-03_(D7), yellow: only INRA C3, pink: only few strains including INRA C3 or F3175-03_(D7)). **D.** Sequences of the recombinant flagellin proteins (rFla) after cloning in the pASK-IBA5+ vector. The putative N-terminal signal peptide for secretion was replaced by a strep-tag and a linker sequence. **E.** Alignment of the bcf_08380 promoter region and its homologues of 20 B. cereus strains. bcf_08380 is highlighted in bold. Green: -35 region, orange: -10 region, yellow: +1 according to [1]. Red: Deviations to these sequences in two strains.
(PDF)

S3 Fig Negative controls show the specificity of the established EIAs. **A.** Highly specific indirect EIAs with mAb 1A11. **B.** Indirect EIAs with rabbit antiserum # 5321. **C.** Highly specific sandwich EIAs. Bt: B. thuringiensis, Bw: B. weihenstephanensis, Bps: B. pseudomycoides, Bs: B. subtilis, Bl: B. licheniformis, Ba: B. amyloliquefaciens, Bpu: B. pumilus, Lm: L. monocytogenes. B. cereus strain F837/76 is shown for comparison. Results indicate means and standard deviations of two biological with three technical replicates for each strain.
(PDF)

## References

[1] Karmakar R. State of the art of bacterial chemotaxis. J Basic Microbiol. 2021; 61(5):366–379. doi: 10.1002/jobm.202000661 33687766

[2] Mukherjee S, Kearns DB. The structure and regulation of flagella in *Bacillus subtilis*. Annu Rev Genet. 2014;48:319–40. doi: 10.1146/annurev-genet-120213-092406 25251856 PMC4869327

[3] Nakamura S, Minamino T. Flagella-driven motility of bacteria. Biomolecules. 2019;9(7):279. doi: 10.3390/biom9070279 31337100 PMC6680979

[4] Zhuang XY, Lo CJ. Construction and Loss of Bacterial Flagellar Filaments. Biomolecules. 2020;10(11):1528. doi: 10.3390/biom10111528 33182435 PMC7696725

[5] Jarrell KF, McBride MJ. The surprisingly diverse ways that prokaryotes move. Nat Rev Microbiol. 2008;6(6):466–76. doi: 10.1038/nrmicro1900 18461074

[6] Terahara N, Namba K, Minamino T. Dynamic exchange of two types of stator units in *Bacillus subtilis* flagellar motor in response to environmental changes. Comput Struct Biotechnol J. 2020;18:2897–907. doi: 10.1016/j.csbj.2020.10.009 33163150 PMC7595845

[7] Bonifield HR, Hughes KT. Flagellar phase variation in *Salmonella enterica* is mediated by a posttranscriptional control mechanism. J Bacteriol. 2003;185(12):3567–74. doi: 10.1128/JB.185.12.3567-3574.2003 12775694 PMC156219

[8] Lövgren A, Zhang MY, Engstrom A, Landen R. Identification of two expressed flagellin genes in the insect pathogen *Bacillus thuringiensis* subsp. *alesti*. J Gen Microbiol. 1993;139(1):21–30. doi: 10.1099/00221287-139-1-21 8383729

[9] Tagawa Y. Isolation and characterization of flagellar filaments from *Bacillus cereus* ATCC 14579. Antonie Van Leeuwenhoek. 2014;106(6):1157–65. doi: 10.1007/s10482-014-0285-2 25227778

[10] Xu D, Cote JC. Sequence diversity of *Bacillus thuringiensis* flagellin (H antigen) protein at the intra-H serotype level. Appl Environ Microbiol. 2008;74(17):5524–32. doi: 10.1128/AEM.00951-08 18586969 PMC2546614

[11] Gohar M, Gilois N, Graveline R, Garreau C, Sanchis V, Lereclus D. A comparative study of *Bacillus cereus*, *Bacillus thuringiensis* and *Bacillus anthracis* extracellular proteomes. Proteomics. 2005;5(14):3696–711. doi: 10.1002/pmic.200401225 16167365

[12] Salvetti S, Faegri K, Ghelardi E, Kolstø AB, Senesi S. Global gene expression profile for swarming *Bacillus cereus* bacteria. Appl Environ Microbiol. 2011;77(15):5149–56. doi: 10.1128/AEM.00245-11 21642396 PMC3147438

[13] Harshey RM, Partridge JD. Shelter in a Swarm. J Mol Biol. 2015;427(23):3683–94. doi: 10.1016/j.jmb.2015.07.025 26277623 PMC4548829

[14] Liaqat I, Mirza SA, Iqbal R, Ali NM, Saleem G, Majid S, et al. Flagellar motility plays important role in Biofilm formation of *Bacillus cereus* and *Yersinia enterocolitica*. Pak J Pharm Sci. 2018;31(5):2047–52. 30393211

[15] Okshevsky M, Greve Louw M, Otero Lamela E, Nilsson M, Tolker‐Nielsen T, Meyer RL. A transposon mutant library of *Bacillus cereus* ATCC 10987 reveals novel genes required for biofilm formation and implicates motility as an important factor for pellicle‐biofilm formation. Microbiol Open. 2017;7(2):e00552. doi: 10.1002/mbo3.552 29164822 PMC5911993

[16] Lewis K. Riddle of biofilm resistance. Antimicrob Agents Chemother. 2001;45(4):999–1007. doi: 10.1128/AAC.45.4.999-1007.2001 11257008 PMC90417

[17] Rütschlin S, Böttcher T. Inhibitors of bacterial swarming behavior. Chemistry. 2020;26(5):964–79. doi: 10.1002/chem.201901961 31268192 PMC7027876

[18] Chaban B, Hughes HV, Beeby M. The flagellum in bacterial pathogens: For motility and a whole lot more. Semin Cell Dev Biol. 2015;46:91–103. doi: 10.1016/j.semcdb.2015.10.032 26541483

[19] Dunne C, Dolan B, Clyne M. Factors that mediate colonization of the human stomach by *Helicobacter pylori*. World J Gastroenterol. 2014;20(19):5610–24. doi: 10.3748/wjg.v20.i19.5610 24914320 PMC4024769

[20] Josenhans C, Suerbaum S. The role of motility as a virulence factor in bacteria. Int J Med Microbiol. 2002;291(8):605–14. doi: 10.1078/1438-4221-00173 12008914

[21] Lertsethtakarn P, Ottemann KM, Hendrixson DR. Motility and chemotaxis in *Campylobacter* and *Helicobacter*. Annu Rev Microbiol. 2011;65:389–410. doi: 10.1146/annurev-micro-090110-102908 21939377 PMC6238628

[22] Jessberger N, Dietrich R, Granum PE, Märtlbauer E. The *Bacillus cereus* food infection as multifactorial process. Toxins (Basel). 2020;12(11):e701. doi: 10.3390/toxins12110701 33167492 PMC7694497

[23] Dietrich R, Jessberger N, Ehling-Schulz M, Märtlbauer E, Granum PE. The food poisoning toxins of *Bacillus cereus*. Toxins (Basel). 2021;13(2):e98. doi: 10.3390/toxins13020098 33525722 PMC7911051

[24] Smith V, Josefsen M, Lindbäck T, Hegna IK, Finke S, Tourasse NJ, et al. MogR Is a Ubiquitous Transcriptional Repressor Affecting Motility, Biofilm Formation and Virulence in *Bacillus thuringiensis*. Front Microbiol. 2020;11:610650. doi: 10.3389/fmicb.2020.610650 33424814 PMC7793685

[25] Guinebretiére MH, Auger S, Galleron N, Contzen M, De Sarrau B, De Buyser ML, et al. *Bacillus cytotoxicus* sp. nov. is a novel thermotolerant species of the *Bacillus cereus* Group occasionally associated with food poisoning. Int J Syst Evol Microbiol. 2013;63(1):31–40. doi: 10.1099/ijs.0.030627-0 22328607

[26] Jessberger N, Kranzler M, Da Riol C, Schwenk V, Buchacher T, Dietrich R, et al. Assessing the toxic potential of enteropathogenic *Bacillus cereus*. Food Microbiol. 2019;84:103276. doi: 10.1016/j.fm.2019.103276 31421762

[27] Kamar R, Gohar M, Jehanno I, Rejasse A, Kallassy M, Lereclus D, et al. Pathogenic potential of *Bacillus cereus* strains as revealed by phenotypic analysis. J Clin Microbiol. 2013;51(1):320–3. doi: 10.1128/JCM.02848-12 23135929 PMC3536244

[28] Ghelardi E, Celandroni F, Salvetti S, Ceragioli M, Beecher DJ, Senesi S, et al. Swarming behavior of and hemolysin BL secretion by *Bacillus cereus*. Appl Environ Microbiol. 2007;73(12):4089–93. doi: 10.1128/AEM.02345-06 17449693 PMC1932716

[29] Bouillaut L, Ramarao N, Buisson C, Gilois N, Gohar M, Lereclus D, et al. FlhA influences *Bacillus thuringiensis* PlcR-regulated gene transcription, protein production, and virulence. Appl Environ Microbiol. 2005;71(12):8903–10. doi: 10.1128/AEM.71.12.8903-8910.2005 16332888 PMC1317475

[30] Ghelardi E, Celandroni F, Salvetti S, Beecher DJ, Gominet M, Lereclus D, et al. Requirement of *flhA* for swarming differentiation, flagellin export, and secretion of virulence-associated proteins in *Bacillus thuringiensis*. J Bacteriol. 2002;184(23):6424–33. doi: 10.1128/JB.184.23.6424-6433.2002 12426328 PMC135439

[31] Mazzantini D, Celandroni F, Salvetti S, Gueye SA, Lupetti A, Senesi S, et al. FlhF is required for swarming motility and full pathogenicity of *Bacillus cereus*. Front Microbiol. 2016;7:1644. doi: 10.3389/fmicb.2016.01644 27807433 PMC5069341

[32] Salvetti S, Ghelardi E, Celandroni F, Ceragioli M, Giannessi F, Senesi S. FlhF, a signal recognition particle-like GTPase, is involved in the regulation of flagellar arrangement, motility behaviour and protein secretion in *Bacillus cereus*. Microbiology. 2007;153(8):2541–52. doi: 10.1099/mic.0.2006/005553-0 17660418

[33] Senesi S, Salvetti S, Celandroni F, Ghelardi E. Features of *Bacillus cereus* swarm cells. Res Microbiol. 2010;161:743–9. doi: 10.1016/j.resmic.2010.10.007 21035546

[34] Mazzantini D, Fonnesu R, Celandroni F, Calvigioni M, Vecchione A, Mrusek D, et al. GTP-dependent FlhF homodimer supports secretion of a hemolysin in *Bacillus cereus*. Front Microbiol. 2020;11:879. doi: 10.3389/fmicb.2020.00879 32435240 PMC7218170

[35] Gao S, Ni C, Huang W, Hao H, Jiang H, Lv Q, et al. The interaction between flagellin and the glycosphingolipid Gb3 on host cells contributes to *Bacillus cereus* acute infection. Virulence. 2020;11(1):769–80. doi: 10.1080/21505594.2020.1773077 32507026 PMC7567440

[36] Jessberger N, Krey VM, Rademacher C, Böhm ME, Mohr AK, Ehling-Schulz M, et al. From genome to toxicity: a combinatory approach highlights the complexity of enterotoxin production in *Bacillus cereus*. Front Microbiol. 2015;6:560. doi: 10.3389/fmicb.2015.00560 26113843 PMC4462024

[37] Jessberger N, Rademacher C, Krey VM, Dietrich R, Mohr AK, Böhm ME, et al. Simulating intestinal growth conditions enhances toxin production of enteropathogenic *Bacillus cereus*. Front Microbiol. 2017;8:627. doi: 10.3389/fmicb.2017.00627 28446903 PMC5388749

[38] Tausch F, Dietrich R, Schauer K, Janowski R, Niessing D, Märtlbauer E, et al. Evidence for complex formation of the *Bacillus cereus* haemolysin BL components in solution. Toxins (Basel). 2017;9(9):288. doi: 10.3390/toxins9090288 28926954 PMC5618221

[39] Schwenk V, Riegg J, Lacroix M, Märtlbauer E, Jessberger N. Enteropathogenic potential of *Bacillus thuringiensis* isolates from soil, animals, food and biopesticides. Foods. 2020;9(10):1484. 10.3390/foods9101484 PMC7603059 33080854

[40] Leenaars M, Hendriksen CF. Critical steps in the production of polyclonal and monoclonal antibodies: evaluation and recommendations. ILAR J. 2005;46(3):269–79. doi: 10.1093/ilar.46.3.269 15953834

[41] Dietrich R, Fella C, Strich S, Märtlbauer E. Production and characterization of monoclonal antibodies against the hemolysin BL enterotoxin complex produced by *Bacillus cereus*. Appl Environ Microbiol. 1999;65(10):4470–4. doi: 10.1128/AEM.65.10.4470-4474.1999 10508077 PMC91595

[42] Dietrich R, Moravek M, Bürk C, Granum PE, Märtlbauer E. Production and characterization of antibodies against each of the three subunits of the *Bacillus cereus* nonhemolytic enterotoxin complex. Appl Environ Microbiol. 2005;71(12):8214–20. doi: 10.1128/AEM.71.12.8214-8220.2005 16332805 PMC1317347

[43] Brameyer S, Plener L, Muller A, Klingl A, Wanner G, Jung K. Outer Membrane Vesicles Facilitate Trafficking of the Hydrophobic Signaling Molecule CAI-1 between *Vibrio harveyi* Cells. J Bacteriol. 2018;200(15):e00740–17. doi: 10.1128/JB.00740-17 29555694 PMC6040191

[44] Böhm ME, Huptas C, Krey VM, Scherer S. Massive horizontal gene transfer, strictly vertical inheritance and ancient duplications differentially shape the evolution of *Bacillus cereus* enterotoxin operons *hbl*, *cytK* and *nhe*. BMC Evol Biol. 2015;15:246. doi: 10.1186/s12862-015-0529-4 26555390 PMC4641410

[45] Darling AC, Mau B, Blattner FR, Perna NT. Mauve: multiple alignment of conserved genomic sequence with rearrangements. Genome Res. 2004;14(7):1394–403. doi: 10.1101/gr.2289704 15231754 PMC442156

[46] Sievers F, Wilm A, Dineen D, Gibson TJ, Karplus K, Li W, et al. Fast, scalable generation of high-quality protein multiple sequence alignments using Clustal Omega. Mol Syst Biol. 2011;7:539. doi: 10.1038/msb.2011.75 21988835 PMC3261699

[47] Waterhouse A, Bertoni M, Bienert S, Studer G, Tauriello G, Gumienny R, et al. SWISS-MODEL: homology modelling of protein structures and complexes. Nucleic Acids Res. 2018; 46:296–303. doi: 10.1093/nar/gky427 29788355 PMC6030848

[48] Hayakawa J, Kondoh Y, Ishizuka M. Cloning and characterization of flagellin genes and identification of flagellin glycosylation from thermophilic *Bacillus* species. Biosci Biotechnol Biochem. 2009;73(6):1450–2. doi: 10.1271/bbb.90092 19502747

[49] Schirm M, Kalmokoff M, Aubry A, Thibault P, Sandoz M, Logan SM. Flagellin from *Listeria monocytogenes* is glycosylated with beta-O-linked N-acetylglucosamine. J Bacteriol. 2004;186(20):6721–7. doi: 10.1128/JB.186.20.6721-6727.2004 15466023 PMC522210

[50] Lecadet MM, Frachon E, Dumanoir VC, Ripouteau H, Hamon S, Laurent P, et al. Updating the H-antigen classification of *Bacillus thuringiensis*. J Appl Microbiol. 1999;86(4):660–72. doi: 10.1046/j.1365-2672.1999.00710.x 10212410

[51] Murakami T, Hiraoka K, Mikami T, Matsumoto T, Katagiri S, Suzuki M. Detection of *Bacillus cereus* flagellar antigen by enzyme-linked immunosorbent assay (ELISA). Microbiol Immunol. 1991;35(3):223–34. doi: 10.1111/j.1348-0421.1991.tb01551.x 1678490

[52] Taylor AJ, Gilbert RJ. *Bacillus cereus* food poisoning: a provisional serotyping scheme. J Med Microbiol. 1975;8(4):543–50. doi: 10.1099/00222615-8-4-543 813000

[53] Terayama T, Shingaki M, Yamada S, Ushioda H, Igarashi H, Sakai S, et al. Incidence of *Bacillus cereus* in commercial foods and serological typing of isolates. J Food Hyg Soc Jpn. 1978;19(1):98–104.

[54] Mikami T, Hiraoka K, Murakami T, Boon-Long J, Matsumoto T, Suzuki M. Detection of common flagella antigen in *Bacillus cereus* by monoclonal antibody. Microbiol Immunol. 1990;34(8):709–14. doi: 10.1111/j.1348-0421.1990.tb01048.x 2126335

[55] Murakami T, Hiraoka K, Mikami T, Matsumoto T, Katagiri S, Shinagawa K, et al. Analysis of common antigen of flagella in *Bacillus cereus* and *Bacillus thuringiensis*. FEMS Microbiol Lett. 1993;107(2–3):179–83. doi: 10.1111/j.1574-6968.1993.tb06027.x 8472901

[56] Xu D, Cote JC. Sequence diversity of the *Bacillus thuringiensis* and *B*. *cereus sensu lato* flagellin (H antigen) protein: comparison with H serotype diversity. Appl Environ Microbiol. 2006;72(7):4653–62. doi: 10.1128/AEM.00328-06 16820457 PMC1489342

[57] Helgason E, Caugant DA, Olsen I, Kolstø AB. Genetic structure of population of *Bacillus cereus* and *B*. *thuringiensis* isolates associated with periodontitis and other human infections. J Clin Microbiol. 2000;38(4):1615–22. doi: 10.1128/JCM.38.4.1615-1622.2000 10747152 PMC86502

[58] Helgason E, Økstad OA, Caugant DA, Johansen HA, Fouet A, Mock M, et al. *Bacillus anthracis*, *Bacillus cereus*, and *Bacillus thuringiensis*—one species on the basis of genetic evidence. Appl Environ Microbiol. 2000;66(6):2627–30. doi: 10.1128/AEM.66.6.2627-2630.2000 10831447 PMC110590

[59] Hoggarth A, Weaver A, Pu Q, Huang T, Schettler J, Chen F, et al. Mechanistic research holds promise for bacterial vaccines and phage therapies for *Pseudomonas aeruginosa*. Drug Des Devel Ther. 2019;13:909–24. doi: 10.2147/DDDT.S189847 30936684 PMC6431001

[60] Salah Ud-Din AIM, Roujeinikova A. Flagellin glycosylation with pseudaminic acid in *Campylobacter* and *Helicobacter*: prospects for development of novel therapeutics. Cell Mol Life Sci. 2018;75(7):1163–78. doi: 10.1007/s00018-017-2696-5 29080090 PMC11105201

