# Supplementary material for: Characterization of strain-specific Bacillus cereus swimming motility and flagella by means of specific antibodies
Source: PLoS One. 2022 Mar 17;17(3):e0265425. doi: 10.1371/journal.pone.0265425 (PMC8929632; doi:10.1371/journal.pone.0265425)
Supplement: S1 Fig — X indicates parts of the images not used for the manuscript figures. Chemiluminescence signals were detected on a UVP ChemStudio imager (Analytik Jena, Jena, Germany) after 20–120 s exposure. Samples on 1% agarose gels were made visible on a UV table and photographed with a Huawei Psmart cell phone. (PDF) [file pone.0265425.s001.pdf]

**Blots Fig 1A**

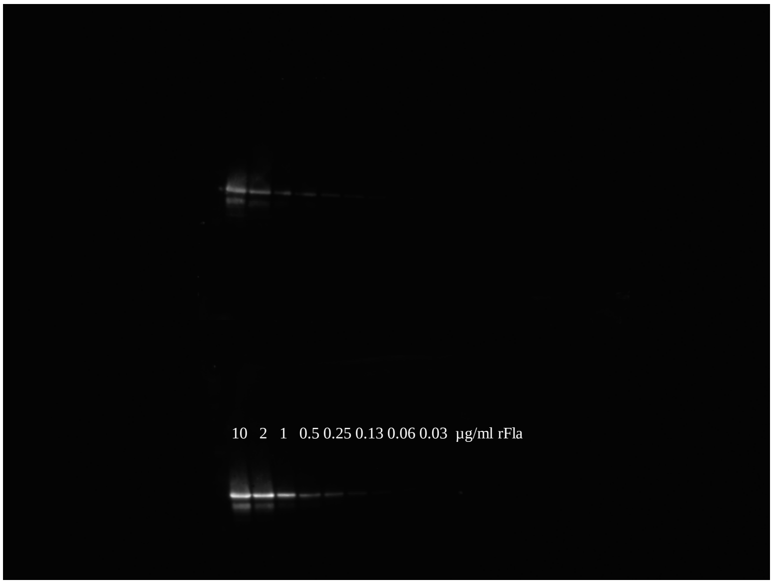


X

The lower blot was used for the 1A11 figure.

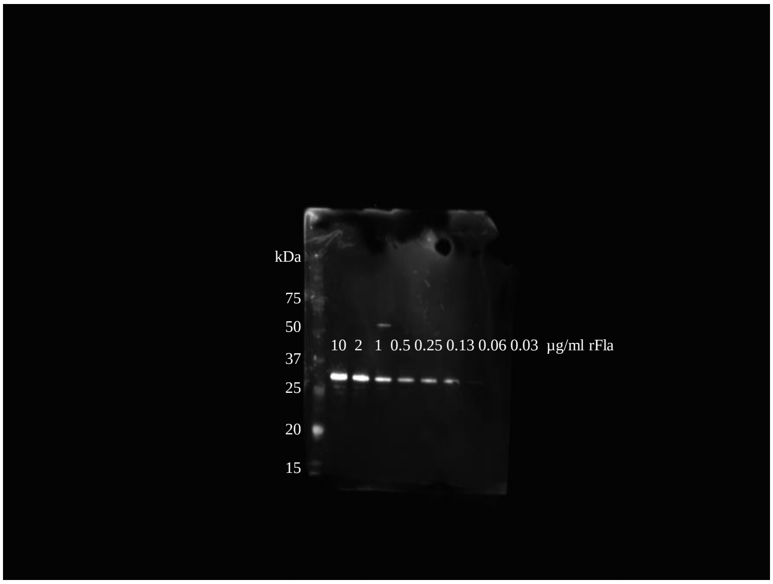


The whole blot was used for the # 5320 figure.

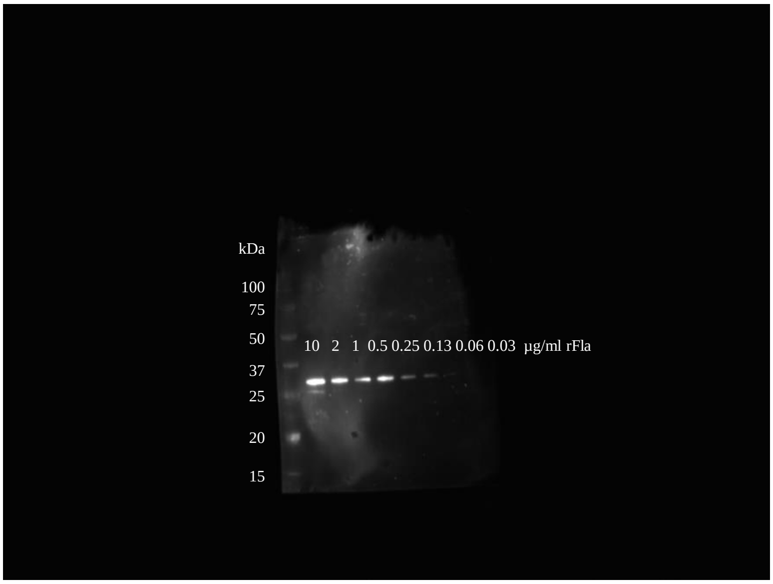


The whole blot was used for the # 5321 figure.

# Blots Fig 1B

The whole blots were used for Fig 1B.

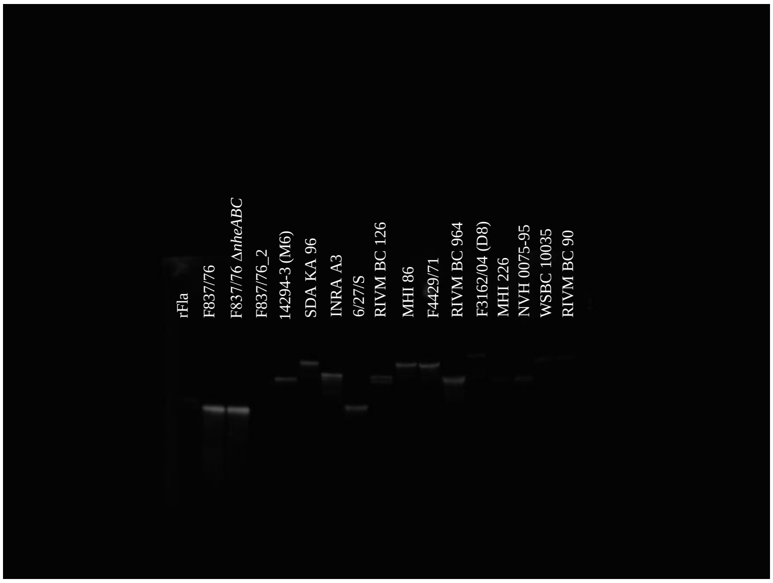


left

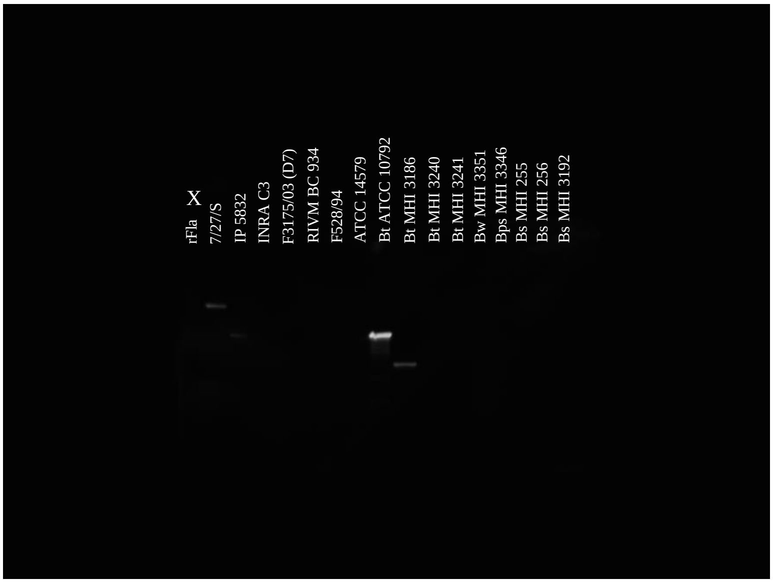


middle

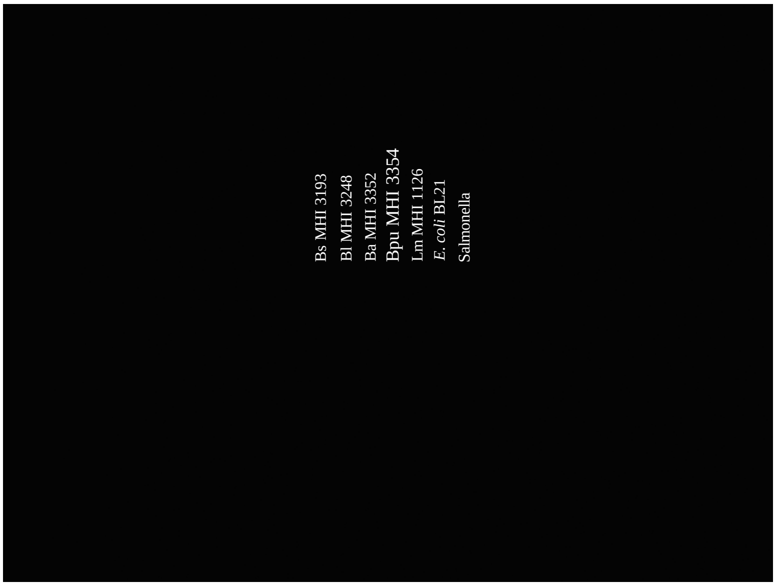


right

# Blots Fig 1C

The whole blots were used for Fig 1C.

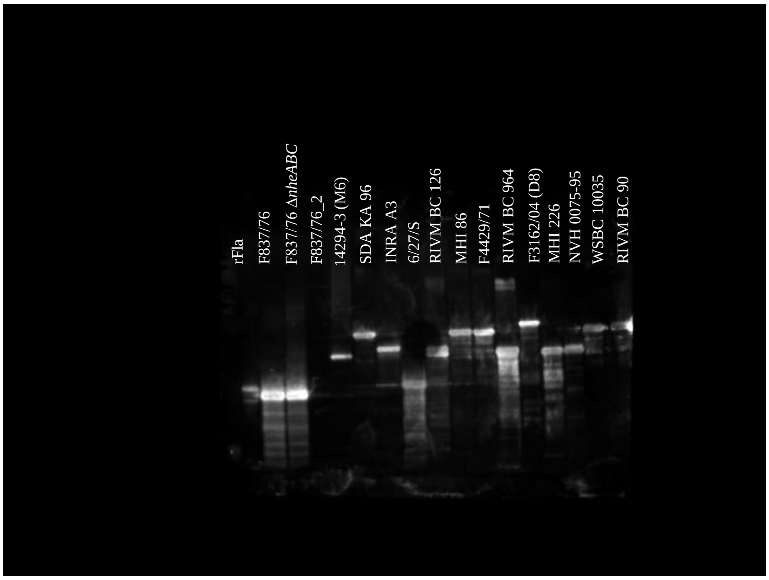


left

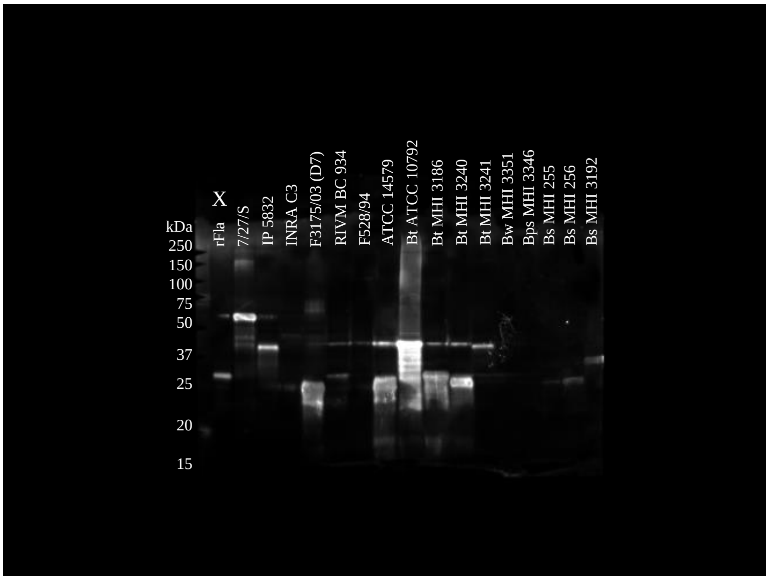


middle

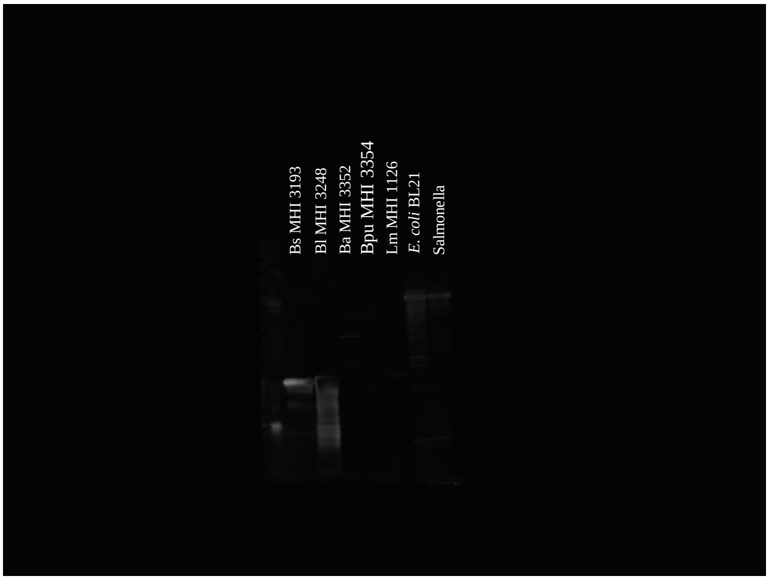


right

# Blot Fig 3C

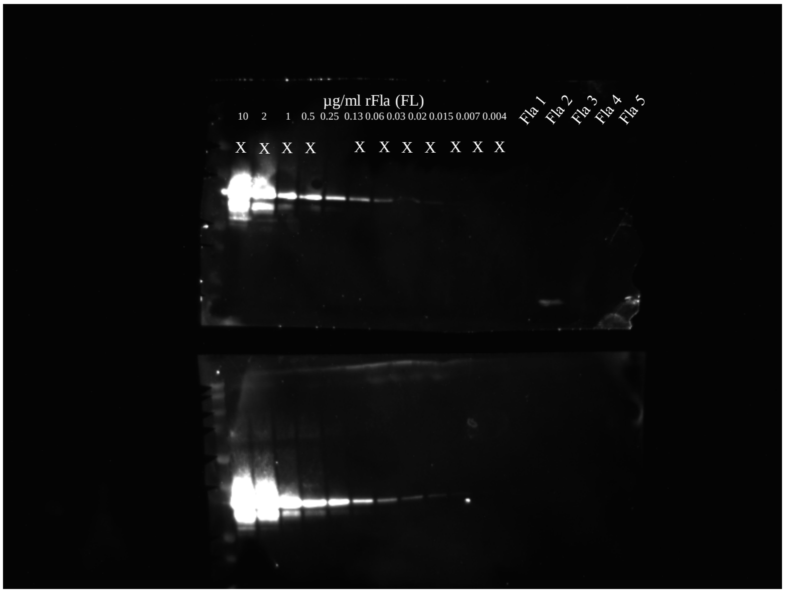


The upper blot was used for the figure.

X

## Gels Fig 4C

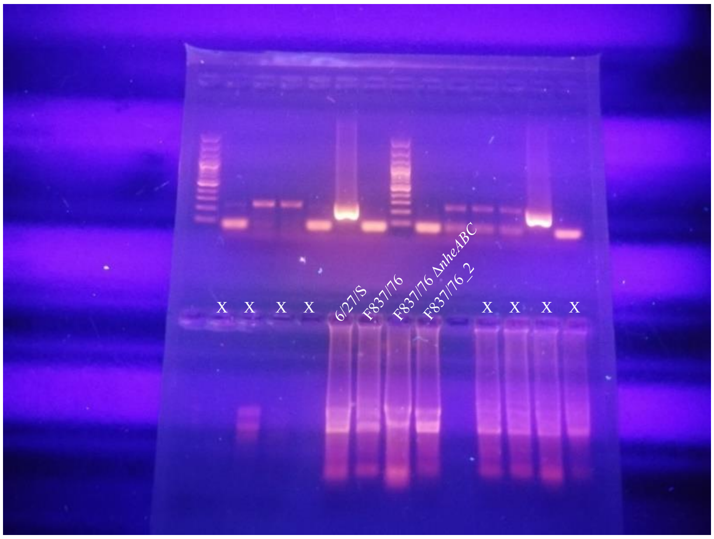


X

The lower part of the gel was used for the image on the left.

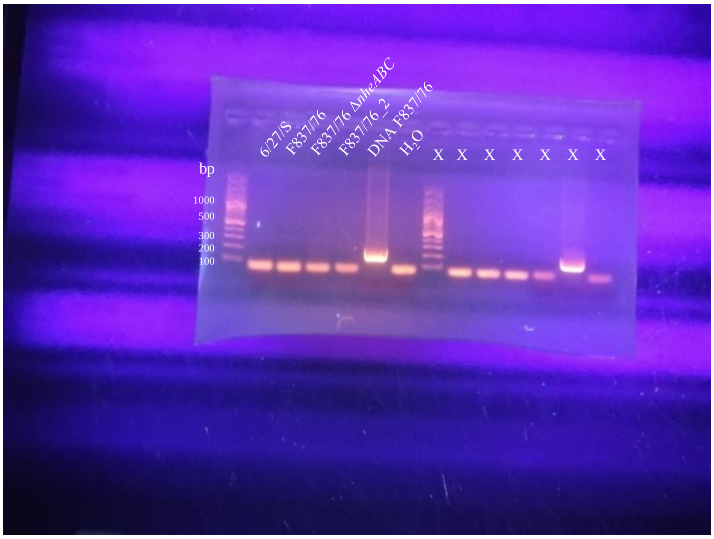


The left part of the gel was used for the image in the middle.

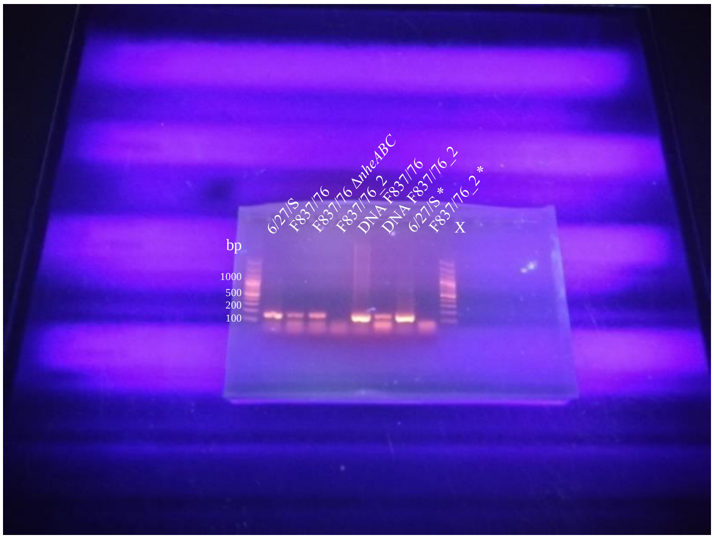


The whole gel was used for the image on the right.

**Fig S1: Uncropped and unadjusted images of blots and gels.** X indicates parts of the images not used for the manuscript figures. Chemiluminescence signals were detected on a UVP ChemStudio imager (Analytik Jena, Jena, Germany) after 20-120 s exposure. Samples on 1 % agarose gels were made visible on a UV table and photographed with a Huawei Psmart cell phone.
