# Supplementary material for: Characterization of strain-specific Bacillus cereus swimming motility and flagella by means of specific antibodies
Source: PLoS One. 2022 Mar 17;17(3):e0265425. doi: 10.1371/journal.pone.0265425 (PMC8929632; doi:10.1371/journal.pone.0265425)
Supplement: S2 Fig — A. Comparison of bcf_08380 (strain F837/76) and the three verified flagellin-encoding genes of strain ATCC 14579. B. Alignment of the corresponding protein sequences. The epitope most likely recognized by mAb 1A11 is marked in green. C. Alignment of the bcf_08380 gene product and its homologues of 19 enteropathogenic and apathogenic B. cereus strains. bcf_08380 is highlighted in bold. The mAb 1A11 epitope is shown in green. Strains INRA C3 and F3175-03_(D7) are underlined. Amino acid substitutions in the N-terminal region are highlighted (red: only strain F3175-03_(D7), yellow: only INRA C3, pink: only few strains including INRA C3 or F3175-03_(D7)). D. Sequences of the recombinant flagellin proteins (rFla) after cloning in the pASK-IBA5+ vector. The putative N-terminal signal peptide for secretion was replaced by a strep-tag and a linker sequence. E. Alignment of the bcf_08380 promoter region and its homologues of 20 B. cereus strains. bcf_08380 is highlighted in bold. Green: -35 region, orange: -10 region, yellow: +1 according to [1]. Red: Deviations to these sequences in two strains. (PDF) [file pone.0265425.s002.pdf]

A. flagellin gene alignment F837/76 and ATCC 14579

```
bcf_08380      ATGAGAATTAATACAAACATTAACAGCATGCGTACTCAAGAGTACATGCGCCAAAACCAA 60
BC_1658        ATGAGAATTAATACAAACATTAACAGCATGCGTACGCAAGAGTACATGCGACAAAACCAA 60
BC_1657        ATGAGAATTAATACAAATATTAATAGTATGCGTACGCAAGAGTACATGCGACAAAACCAA 60
BC_1659        ATGAGAATTAATACAAACATTAACAGCATGCGTACGCAAGAGTACATGCGACAAAACCAA 60
               ***************** ***** ** ******** ************** *********

bcf_08380      GAAAAAATGAATACAGCTATGAACCGTTTATCAAGCGGTAAACGCATCAATAGTGCTGCG 120
BC_1658        GACAAAATGAATACTGCGATGAATCGTTTATCTAGCGGTAAATCTATTAACAGTGCAGCT 120
BC_1657        GACAAAATGAATACTGCGATGAATCGTTTATCTAGCGGTAAATCTATTAACAGTGCAGCT 120
BC_1659        GACAAAATGAATACTGCGATGAATCGTTTATCTAGCGGTAAATCTATTAACAGTGCAGCT 120
               ** *********** ** ***** ******** *********   ** ** ***** **

bcf_08380      GATGATGCAGCGGGTCTTGCTATTGCTACTCGTATGCGTGCACAAGAAAGCGGGTTAAAT 180
BC_1658        GACGATGCGGCTGGTTTAGCTATCGCTACTCGTATGCGTGCAAAAGAAGGCGGATTAAAC 180
BC_1657        GACGATGCGGCTGGTTTAGCTATCGCTACTCGTATGCGTGCAAAAGAAGGCGGATTAAAC 180
BC_1659        GACGATGCGGCTGGTTTAGCTATCGCTACTCGTATGCGTGCAAAAGAAGGCGGATTAAAC 180
               ** ***** ** *** * ***** ****************** ***** **** *****

bcf_08380      GTCGCGTCTCGTAATACTCAAGACGGTATCTCTTTAATTCAAACAGCTGATTCTGCATTA 240
BC_1658        GTCGGAGCACGTAACACGCAAGACGCTATGTCTGCTTTACGTACTGGTGACGCTGCTTTG 240
BC_1657        GTCGGAGCACGTAACACGCAAGACGCTATGTCTGCTTTACGTACTGGTGACGCTGCTTTG 240
BC_1659        GTCGGAGCACGTAACACGCAAGACGCTATGTCTGCTTTACGTACTGGTGACGCTGCTTTG 240
               ****   * ***** ** ******* *** ***    * *  ** * ***  **** **

bcf_08380      GGCACAGTATCTAACATTTTACATCGTATGCGTGACCTTGCTGTTCAATCTGCTAACGGC 300
BC_1658        GGATCTATTTCTAACATCTTACTTCGTATGCGTGACCTTGCTACACAAGCTGCAAACGGT 300
BC_1657        GGATCTATTTCTAACATCTTACTTCGTATGCGTGACCTTGCTACACAGGCTTCTAACGGT 300
BC_1659        GGATCTATTTCTAACATCTTACTTCGTATGCGTGACCTTGCTACACAGGCTTCTAACGGT 300
               **  *  * ******** **** *******************   **  ** * *****

bcf_08380      TCAAATGGAGAAACAGATCGTGACGCTTTAGATGGTGAATACCAACAGCTAATTACCCAG 360
BC_1658        ACGAATAACGCTGAAGATACTGCCTCTTTAGACAAAGAGTATGTTGCCTTAAAAGATGAA 360
BC_1657        ACGAATAACACTGAAGATACTGCTTCTTTAAACAAAGAATACGAAGCATTAAAAAGTGAA 360
BC_1659        ACGAATAACACTGAAGATACTGCTTCTTTAAACAAAGAATACGAAGCATTAAAAAGTGAA 360
                * ***        ****  **   ***** *    ** **        ***      *

bcf_08380      ATTGGATACATTGCAGATAATACAGAATTTAATGGAACGAATCTGTTAAAAGCTGATACT 420
BC_1658        ATTGATCACATTGCTGGAAAAACGAATTTTAACGGTAATTCTTTCTTAGATACAACTGCC 420
BC_1657        ATTGATCATATCGCTGACAAAACATCTTTCAATGGTATTAAATTCTTAAGTACTGATA-- 418
BC_1659        ATTGATCATATCGCTAACAAAACATCTTTCAATGGTATTAAATTCTTAAGTACTGATA-- 418
               ****   * ** **    ** **    ** ** ** *      * ***    *   *

bcf_08380      ACAATTTCCATCAAATCTACTGATAAATCAAAAGCT------------AGTACAATTGAC 468
BC_1658        ACTCCTCCCGGAAAAGATATCGAGATTCAACTTTCTGATGCTTCTGGTGATACAATGACA 480
BC_1657        -AGGGTGGTGCGGCTGATATCAAAGTTCAACTTTCTGATGCTTCTGGCGATACAATGACA 477
BC_1659        -AGGGTGGTGCGGCTGATATTAAAGTTCAACTTTCTGATGCTTCTGGCGATACAATGACA 477
                    *           **   *      *    **              ******

bcf_08380      ATCGAAACT---------AAAGCAGTTGACGGAAGTGGTTTAGGTAGTATTACAGGTACT 519
BC_1658        CTCAAAGCCATTGATACAAAATCATTGACAACTGGCACTTTAACTAATTTA------AAA 534
BC_1657        ATTGATTCTCTGAATGCAAAAACAATTACAACTGATACTTTAACTACTTTA------GCA 531
BC_1659        ATTGACTCTCTAGATGCAAAGAAAATTACAACAGCTAATCTAACTACTTTA------GCA 531
                *  *  *          **   * *            * **  ** * *

bcf_08380      GATGGTACTACAGCAGATGAGGCAATTGAAACAATAGATACAATGATAGATGCAATTGCA 579
BC_1658        GATAGAGCCACTGCTGAAACTGAAATAACAAAACTGGACACTGCAATTCAAAAAATTGCT 594
BC_1657        GATAGAGCCACTGCTGAAACTGAAATAACAAAACTGGACACTGCAATTCAAAAAATTGCT 591
BC_1659        GACAGAGGAACTGCTGAGACTGAAATAAAAGCTCTTGACACTGCTATCCAAGAACTAGCT 591
               **  *    ** ** **    * ***   *    * ** **    **  *   * * **

bcf_08380      GGAAACCGAGCTACATTAGGTGCTACATTAAATCGTTTACAATATAATGCCGAAAACTTA 639
BC_1658        GATGAAAGAGCAACTTTCGGTTCTCAATTAAACCGTTTAGACCATAACTTAAACAACGTA 654
BC_1657        GATGAAAGAGCAACTTTCGGTTCTCAATTAAACCGTTTAGACCATAACTTAAACAACGTA 651
BC_1659        GACGCTAGAGCAACTTTCGGTTCTCAATTAAACCGTTTAGACCATAACTTAAACAACGTA 651
               *      **** ** ** *** **  ****** ****** *  ****     * *** **

bcf_08380      AACAATCAATCTACAGCTATGTCATCTTCAGCTTCTCAAATTGAAGATGCTGATATGGCA 699
BC_1658        ACGAGCCAAGCTACTAACATGGCAGCAGCTGCTTCTCAAATCGAAGATGCTGATATGGCA 714
BC_1657        ACAAGCCAAGCTACTAACATGGCTTCAGCTGCTTCTCAAATCGAAGACGCTGACATGGCG 711
BC_1659        ACGAGCCAAGCTACTAACATGGCAGCAGCTGCTTCTCAAATCGAAGATGCTGATATGGCA 711
               *  *  *** ****    *** *  *  * *********** ***** ***** *****

bcf_08380      AAAGAAATGTCTGAAATGACTAAGTTCA--AAATCTTGAACGAAGCTGGCATCAGCATGC 757
BC_1658        AAAGAGATGTCTGAAATGACTAAGTTCA--AAATTTTAAACGAAGCTGGTATCAGCATGC 772
BC_1657        AAAGAAATGTCTGAAATGACTAAGTTCA--AAATCTTGAATGAAGCTGGTATCAGCATGC 769
BC_1659        AAAGAGATGTCTGAAATGACTAAGGTTCAAAATCCTTGAACGAAGCTGGTATCAGCATGC 771
               ***** ****************** *    **   ** ** ******** **********
```

```
bcf_08380      TTTCTCAAGCAAACCAAACTCCACAAATGGTAAGTAAATTATTACAATAA  807
BC_1658        TTTCTCAAGCTAACCAAACACCACAAATGGTTTCTAAATTATTACAATAA  822
BC_1657        TTTCTCAAGCAAACCAAACTCCACAAATGGTTTCCAAATTATTACAATAA  819
BC_1659        TTTCTCAAGCTAACCAAACACCACAAATGGTTTCTAAATTATTACAATAA  821
               ********** ******** ***********    ***************
```

B. flagellin protein alignment F837/76 and ATCC 14579

```
bcf_08380      MRINTNINSMRTQEYMRQNQEKMNTAMNRLSSGKRINSAADDAAGLAIATRMRAQESGLN  60
BC_1659        MRINTNINSMRTQEYMRQNQDKMNTAMNRLSSGKSINSAADDAAGLAIATRMRAKEGGLN  60
BC_1657        MRINTNINSMRTQEYMRQNQDKMNTAMNRLSSGKSINSAADDAAGLAIATRMRAKEGGLN  60
BC_1658        MRINTNINSMRTQEYMRQNQDKMNTAMNRLSSGKSINSAADDAAGLAIATRMRAKEGGLN  60
               ********************:************* *******************:*.***

bcf_08380      VASRNTQDGISLIQTADSALGTVSNILHRMRDLAVQSANGSNGETDRDALDGEYQQLITQ  120
BC_1659        VGARNTQDAMSALRTGDAALGSISNILLRMRDLATQASNGTNNTEDTASLNKEYEALKSE  120
BC_1657        VGARNTQDAMSALRTGDAALGSISNILLRMRDLATQASNGTNNTEDTASLNKEYEALKSE  120
BC_1658        VGARNTQDAMSALRTGDAALGSISNILLRMRDLATQAANGTNNAEDTASLDKEYVALKDE  120
               *.:*****.:* ::*.*:***::**** ******.*::**:*.  *  :*: **  *  :

bcf_08380      IGYIADNTEFNGTNLLKADTTISIKST-----DKSKASTID---IETKAVDGSGLGSITG  172
BC_1659        IDHIANKTSFNGIKFLSTDK-GGAADIKVQLSDASG-DTMTIDSLDAKKITTANLT--TL  176
BC_1657        IDHIADKTSFNGIKFLSTDK-GGAADIKVQLSDASG-DTMTIDSLNAKTITTDTLT--TL  176
BC_1658        IDHIAGKTNFNGNSFLDTTATPPGKDIEIQLSDASG-DTMTLKAIDTKSLTTGTLT--NL  177
               *.:**.:*.*** .:*.:        .      * *  .*:    :::* :    *   .

bcf_08380      TDGTTADEAIETIDTMIDAIAGNRATLGATLNRLQYNAENLNNQSTAMSSSASQIEDADM  232
BC_1659        ADRGTAETEIKALDTAIQELADARATFGSQLNRLDHNLNNVTSQATNMAAAASQIEDADM  236
BC_1657        ADRATAETEITKLDTAIQKIADERATFGSQLNRLDHNLNNVTSQATNMASAASQIEDADM  236
BC_1658        KDRATAETEITKLDTAIQKIADERATFGSQLNRLDHNLNNVTSQATNMAAAASQIEDADM  237
                *  **:  *  :** *: :*. ***:*: ****::* :*:..*:* *:::*********

bcf_08380      AKEMSEMTKFKILNEAGISML--SQANQTPQMVSKLLQ    268
BC_1659        AKEMSEMTKVQNP-TKLVSACFLKLTKHHK-WFLNYYN    272
BC_1657        AKEMSEMTKFKILNEAGISML--SQANQTPQMVSKLLQ    272
BC_1658        AKEMSEMTKFKILNEAGISML--SQANQTPQMVSKLLQ    273
               *********.:      :*    . :::    . :  :
```

C. flagellin protein alignment of 20 strains

```
14294-3_(M6)      MRINTNINSMRTQEYMRQNQAKMSNAMDRLSSGKRINNASDDAAGLAIATRMRARESGLG      60
SDA_KA96          MRINTNINSMRTQEYMRQNQAKMSNAMDRLSSGKRINNASDDAAGLAIATRMRARESGLG      60
INRA_A3           MRINTNINSMRTQEYMRQNQAKMSNSMDRLSSGKRINNASDDAAGLAIATRMRSREGGLN      60
INRA_C3           MRINTNINSMRTQEYMRQNQTKMSNAMDRLSSGKRINNASDDAAGLAIATRMRARENGLG      60
6/27/S            MRINTNINSMRTQEYMRQNQDKMNNAMNRLSSGKRINSAADDAAGLAIATRMRAKESGLN      60
F3175-03 (D7)     MRINTNINSMRTQEYMRQNQDKMNTAMNRLSSGKSINSAADDAAGLAIATRMRAKEGGLN      60
RIVM_Bc934        MRINTNINSMRTQEYMRQNQDKMNNAMNRLSSGKRINSAADDAAGLAIATRMRAKESGLN      60
F528/94           MRINTNINSMRTQEYMRQNQDKMNTSMNRLSSGKRINSAADDAAGLAIATRMRARESGLG      60
bcf_08380         MRINTNINSMRTQEYMRQNQEKMNTAMNRLSSGKRINSAADDAAGLAIATRMRAQESGLN      60
RIVM_Bc126        MRINTNINSMRTQEYMRQNQAKMSNAMDRLSSGKRINNASDDAAGLAIATRMRARESGLN      60
MHI86             MRINTNINSMRTQEYMRQNQAKMSNAMDRLSSGKRINNASDDAAGLAIATRMRARESGLN      60
F4429/71          MRINTNINSMRTQEYMRQNQAKMSNAMDRLSSGKRINNASDDAAGLAIATRMRARESGLN      60
RIVM_Bc964        MRINTNINSMRTQEYMRQNQAKMSNAMDRLSSGKRINNASDDAAGLAIATRMRARESGLG      60
F3162/04_(D8)     MRINTNINSMRTQEYMRQNQAKMSNAMDRLSSGKRINNASDDAAGLAIATRMRARESGLG      60
MHI226            MRINTNINSLRTQEYMRQNQSKMSNSMDRLSSGKRINNASDDAAGLAIATRMRARESGLN      60
NVH_0075-95       MRINTNINSMRTQEYMRQNQAKMSNAMDRLSSGKRINNASDDAAGLAIATRMRARESGLG      60
WSBS10035         MRINTNINSMRTQEYMRQNQAKMSTAMDRLSSGKRINNASDDAAGLAIATRMRSRESGLG      60
RIVM_Bc90         MRINTNINSMRTQEYMRQNQAKMSTAMDRLSSGKRINNASDDAAGLAIATRMRSRESGLG      60
7/27/S            MRINTNINSMRTQEYMRQNQAKMSNAMDRLSSGKRINNASDDAAGLAIATRMRARESGLS      60
IP5832            MRINTNINSMRTQEYMRQNQAKMSNAMDRLSSGKRINNASDDAAGLAIATRMRARESGLG      60
                  *********:********** **..:*:****** **.*:*************::* **

14294-3_(M6)      VAANNTQDGMSVIRTADSALGSVSNILLRMRDIANQSANGTNTLENQGALNKEFDALKEQ      120
SDA_KA96          VAANNTQDGMSLIRTADSALNSVSNILLRMRDIANQSANGTNTTDNQKALDKEFSALKEQ      120
INRA_A3           VAARNTEDGMSLIRTADSALNSVSNILLRMRDLANQSASGTNTDKNQAAMQKEFDQLKEQ      120
INRA_C3           VAANNTQDGMSLIRTADSAMNSVSNILLRMRDLANQSANGTNTDDNQKALDKEFSALKEQ      120
6/27/S            VAARNTQDGMSLIRTADSALDSVSNILLRMRDLANQSATGSNNKGNQASLQKEFAELQDQ      120
F3175-03 (D7)     VGARNTQDAMSALRTGDAALGSISNILLRMRDLATQAASGTNNVKDSASLNKEYQQLAKE      120
RIVM_Bc934        VAARNTQDGMSLIRTADSALDSVSNILLRMRDLANQSATGSNNKGNQASLQKEFAELQDQ      120
F528/94           KAAQNTQDGMSLIRTAESALNSVSNILTRMRDLAVQSSSGANNSDNQEALQKEFKALQEQ      120
bcf_08380         VASRNTQDGISLIQTADSALGTVSNILHRMRDLAVQSANGSNGETDRDALDGEYQQLITQ      120
RIVM_Bc126        VAANNTQDGISLIRTADSALNSVSNILLRMRDIANQSANGTNTDTNQAALQKEFGELQKQ      120
MHI86             VAANNTQDGMALIRTADSAMNSVSNILLRMRDLANQSANGTNTDKNQAALQKEFSELQKQ      120
F4429/71          VAANNTQDGMALIRTADSAMNSVSNILLRMRDLANQSANGTNTDKNQAALQKEFSELQKQ      120
RIVM_Bc964        VAANNTQDGISLIRTADSAMNSVSNILLRMRDLANQSANGTNTNENQAALNKEFDALKEQ      120
F3162/04_(D8)     VAANNTQDGMALIRTADSAMNSVSNILLRMRDIANQSANGTNTDKNQAALQKEFGELQKQ      120
```

```
MHI226               VAANNTQDGMSLIRTADSAMNSVSNILLRMRDIANQSANGTNTDSNKSALQKEFVELQKQ    120
NVH_0075-95          VASNNTQDGMSLIRTADSALNSVSNILLRMRDLANQSANGTNTNENKAAMQKEFGELKEQ    120
WSBS10035            VAANNTQDGMSLIRTADSALGSVSNILLRMRDLANQSANGTNTNKNQVALQKEFAELQKQ    120
RIVM_Bc90            VAANNTQDGMSLIRTADSALGSVSNILLRMRDLANQSANGTNTNKNQVALQKEFAELQKQ    120
7/27/S               VAADNTQNGMSLIRTADSAMNSVSNILLRMRDIANQSANGTNTDKNQVALQKEFAALKEQ    120
IP5832               VAANNTQDGISLIRTADSAMNSVSNILLRMRDLANQSANGTNTSENQAALDKEFGALKEQ    120
                      .: **::.:: ::*.::*: ::**** ****:* *::.*:*   :  ::: *:  *  :

14294-3_(M6)         IDYIAKNTQFNDKNLLDG---SKASISIQTLDSSETSKQININLANVSTSALKIDTLTIA    177
SDA_KA96             IDYISKNTEFNDKKLLNG---QNATIAIQTLDSADTNKQININLADTSTSALNINNLSIA    177
INRA_A3              IQYIADNTEFNDKKLLDG---SNNTINIQTLDSHDKTKQITISLDSASLKNLDIKDLAIG    177
INRA_C3              IDYISKNTEFNDKKLLNG---ENKTIAIQTLDNADTTKQININLADSSTSALQIDKLTIS    177
6/27/S               IGYIAKNTQFNDQSLLSG---D-KEVAIQTIDSSDATIQLKIQLKDSTLTGLKLEKATVK    176
F3175-03_(D7)        IDHIAEKTNFNGNSFLNQ---A-GGG----------------------------------    142
RIVM_Bc934           IGYIAKNTQFNDQSLLSG---D-KEVAIQTIDSSDATIQLKIQLKDSTLTGLKLEKATVK    176
F528/94              IDYISKNTEFNDQKLLNG---ATTKIAIETLDGTSTAQQINIGLKNSSTSALQINSSSIS    177
bcf_08380            IGYIADNTEFNGTNLLKA---DT-TISIKSTD---KSKASTIDIETKAVDGSGLGS----    169
RIVM_Bc126           IDYISNNTQFNDKKLLNG---DSSTINIQTLDSDDKTKQIGIELSNTSIQALGIKDLKIG    177
MHI86                IDYISSNTQFNDKNLLDG---SNKTINIQTLDSEDGSRQIGIELGSASTKALGIDTLNIG    177
F4429/71             IDYISSNTQFNDKNLLDG---SNKTINIQTLDSEDGSRQIGIELGSASTKALGIDTLNIG    177
RIVM_Bc964           IDYISTNTEFNDKKLLDG---SNKTIAVQTLDNADTSKQININLSNVSTKELGLDTLSIG    177
F3162/04_(D8)        IDYIAGNTQFNDKNLLDG---SNPSISIQTLDSADQSKQISIDLKSATLEALGIKDLTVG    177
MHI226               ITYIADNTQFNDKNLLKE---D-SAVKIQTLDSSSAAQQIGIDLKGVTLDKLGIDKISIG    176
NVH_0075-95          IKYIAENTQFNDQHLLNADKGITKEIAIQTLDSDSDSKQIKIKLQGSSLEALDIKDLQIG    180
WSBS10035            IDYISSNTQFNDKNLLDG---SNKTINIQTLDSEDGSRQIGIELGSASTKALGIDTLSIG    177
RIVM_Bc90            IDYISSNTQFNDKNLLDG---SNKTINIQTLDSEDGSRQIGIELGSASTKALGIDTLSIG    177
7/27/S               ITYIADNTQFNDKNLLNG---N-QTINIQTLDSHDSTKQIGIDLKSATLEALGIKDLTVG    176
IP5832               INYISTNTEFNDKKLLDG---SNETIAIQTLDNADEGKKIDIKLANVSTQSLSIDKLTIG    177
                     * :*: :*:**   :*.

14294-3_(M6)         GTNSTTLQAADL-----KVATDAAQALQDLDPAK--------------------------    206
SDA_KA96             ASGSSAPLVGANAG-KITTANPLAQALVDFDGTATGKDANERTTGVNNFKAAFDDIKGGL    236
INRA_A3              SNTVNKNDLETLDN-SMKRLEK----------PA--TD----------------------    202
INRA_C3              GKTTDTTKTETIT---V-TDDEIKAA----------------------------------    199
6/27/S               IGE---------------------------------------------------------    179
F3175-03_(D7)        ------------------------------------------------------------    142
RIVM_Bc934           IGE---------------------------------------------------------    179
F528/94              T-----------------------------------------------------------    178
bcf_08380            ------------------------------------------------------------    169
RIVM_Bc126           AQAPDLTELKD-AFTNYKTLKDATSPA--TAAPALAKAKAEAKAAYDKLATDFVNIKDSL    234
MHI86                SSKITEATKDH-ATDTIGGLTSGSD-----------------DKAVALAKKSFDQIKGAI    219
F4429/71             SSKITEATKDH-ATDTIGGLTSGSD-----------------DKAVALAKKSFDQIKGAI    219
RIVM_Bc964           TDKVEKTVYEATAT-SFDTL----------------------------------------    196
F3162/04_(D8)        ATENTLAKATITAKDAFDAAKDASDAA--KKE-IDAAAKDTPSKNDAQLAKEYIEAKATL    234
MHI226               GAATTAIEQTD----------------------IDA------VTN---------------    193
NVH_0075-95          NTELAQKDLDLLN-----------ATMDRLDATVPG------------------------    205
WSBS10035            STKVTQAAIDN-ADDTIKKLDTGSS-----------------ADEVAKAKKAFDEIKDAL    219
RIVM_Bc90            STKVTQAAIDN-ADDTIKKLDTGSS-----------------ADEVAKAKKAFDEIKDAL    219
7/27/S               AVGSTEAKSFADARDALAANQKATEF-------IDA----KTALDGNAVAKAYVEAKTAF    225
IP5832               GASQK--TIDDVAG-KFTAL----------------------------------------    194


14294-3_(M6)         ------------------------------------------------------------    206
SDA_KA96             STEDAQKIYDAIDKFNGSKS-LA-------------------DAQEIGNLYQSMTLAKDT    276
INRA_A3              ------------------------------------------------------------    202
INRA_C3              --------KTDIDEFNDAKKALA-------------------DL----------------    216
6/27/S               ---------------------------------------------------DKITDA---    185
F3175-03_(D7)        ------------------------------------------------------------    142
RIVM_Bc934           ---------------------------------------------------DKITDA---    185
F528/94              ------------------------------------------------------------    178
bcf_08380            ------------------------------------------------------------    169
RIVM_Bc126           SAASTTAVGTT-------------------YPAA--------------------------    249
MHI86                SNADAQALAQAFENYDNAASGEKGA-----------------KAAEIKNVYDTITNFSMA    262
F4429/71             SNADAQALAQAFENYDNAASGEKGA-----------------KAAEIKNVYDTITNFSMA    262
RIVM_Bc964           ------------------------------------------------------------    196
F3162/04_(D8)        AT---------------------LKPTDATYAAKAAEL----------------DAATTA    257
MHI226               -----------------------------GIGALTKDSKVATDIKAIKDSFDKIKAG---    221
NVH_0075-95          ------------------------------------------------------------    205
WSBS10035            TDADSRELAKAFDNYDNASTGSKGT-----------------AAEAIATAYKKIATPATA    262
RIVM_Bc90            TDADSRELAKAFDNYDNASTGSKGT-----------------AAEAIATAYKKIATPATA    262
7/27/S               DA-ASDETKQLVTDYENAKSAAEATPTDSGLADTANEKKLAMEANTVAKTYFEAKAAHDS    284
IP5832               ------------------------------------------------------------    194


14294-3_(M6)         ---------------------ATSKETEDAIAEFKKGFDLVKDYMTEQQV-TDVQQKLDK    244
SDA_KA96             DVTTTKNLATTLTSLDGITTGKTAGDRTTAVNNFETEFNKIKGGMSKEDA-DKITAAIQN    335
INRA_A3              -----------------------GAVQAQDVKDAKTAFDKVKSVYSAADV-EKMNDVFKA    238
INRA_C3              ----------KAETGAGKGDGSTDDEIKTAVSNFTKSFEKIQKFMNDSDI-KTVQTEIEK    265
6/27/S               ------------------------------------------------------------    185
F3175-03_(D7)        ------------------------------------------------------------    142
```

```
RIVM_Bc934        ------------------------------------------------------------     185
F528/94           ------------------------------------------------------------     178
bcf_08380         ------------------------------------------------------------     169
RIVM_Bc126        ------------------------------------------------------------     249
MHI86             KVSEDVYTD-AKTAIKGLKA--TS---------TGKEVAAAREHFDK----------IKD     300
F4429/71          KVSEDVYTD-AKTAIKGLKA--TS---------TGKEVAAAREHFDK----------IKD     300
RIVM_Bc964        ----------------GKTTDATKAALDPQVNATMKEFEKVKSFMSADDV-KKIEAKLDA     239
F3162/04_(D8)     -----LNDN-AKVLVDGYEK--KLTTTKT----KEAEYTAAKEQSTKSTAADLVTKYET     305
MHI226            --------------------------MDA---KDVTAIETALNGFKDGEATP--------     244
NVH_0075-95       -------------------------TRDVDVQAAKDAFDKVKGFYTNSDSVKAIERAFED     240
WSBS10035         KVSKEVYTE-AADVIKGLTD--KS---------TDTEVKAAQEQFNK----------IKD     300
RIVM_Bc90         KVSKEVYTE-AADVIKGLTD--KS---------TDTEVKAAQEQFNK----------IKD     300
7/27/S            -----ASPE-TQGIVTKYNT--KLAALDDAANKAISNFDTAKAAFNESPAAKELVKTMDD     336
IP5832            ----------------NTTTTTDKADIQKEVDAAMKEFDKVKGSMSAADA-KAVTDKLAD     237


14294-3_(M6)      YTV---------------------------DKTV-PNAQEIGAAFVAPT----------A     266
SDA_KA96          FKKAD-G----------------------TGNTL-ENAKEIGNLFTQA------------     359
INRA_A3           YDKAL-ANPLADA--------------TANEAALKAAAENINKEFAKLTKPT--------     275
INRA_C3           FD----A----------------------AAPAL-DKAKGMGTAFTSAMDPK-------A     291
6/27/S            -------------------------------------------------------A----     186
F3175-03_(D7)     ------------------------------------------------------------     142
RIVM_Bc934        -------------------------------------------------------A----     186
F528/94           ------------------------------------------------------------     178
bcf_08380         ------------------------------------------------------------     169
RIVM_Bc126        ------------------------------------------------------------     249
MHI86             YLSKPNRTALENAFKA----FDGTDTTAGTSG-------KQGSDI-----ASKFPATDLS     344
F4429/71          YLSKPNRTALENAFKA----FDGTDTTAGTSG-------KQGSDI-----ASKFPATDLS     344
RIVM_Bc964        YTKAD-D----------------------AGSK--VAAQNLGKEFATLTKLE--------     266
F3162/04_(D8)     AKSNALGNDIAKEYLEAKTAYEANKNDISSKSRFEAAETELNKDITANKAAKVLVETYEK     365
MHI226            ---------------------AAAGVDA-IQAALAGAK----------------LP----     262
NVH_0075-95       YATAS-T----------------------AGTAKADAATAIKAAFDLAANKV------GK     271
WSBS10035         YLSKTQRNEIQKAFND----YNPGGTPPVKAG-------TIATAF-----NTQVT-AAPD     343
RIVM_Bc90         YLSKTQRNEIQKAFND----YNPGGTPPVKAG-------TIATAF-----NTQVT-AAPD     343
7/27/S            AKQAATQNNTANAYLVAKAAAEAAPTDADKQAALENATKAL----EKDDTAKGLVKTYEN     392
IP5832            YNNAA-D----------------------TDVAKAAAAKALGGAFDKT------------     262

14294-3_(M6)      ----GAKANVSANSAIKSIDSALKSIADNRATLGATLNRLDFNVNNLKSQSASMASAASQ     322
SDA_KA96          ----AAGTSYNASNAIKSIDKALETIASNRATLGATLNRLDFNVNNLKSQSASMASAASQ     415
INRA_A3           ------GAAFDPSSAVEKIDQAIEKIASSRATLGATLNRLDFNVTNLKSQENSMAASASQ     329
INRA_C3           GTITKAATRQNASDAIKSIDAALETIASNRATLGATLNRLDFNVNNLKSQSSSMAAAASQ     351
6/27/S            -DAANAADATNATGAIKAIDDALRTVAENRATLGATLNRFDFNVNNLKSQESTMASSASQ     245
F3175-03_(D7)     ------------------------------------------------------------     142
RIVM_Bc934        -DAANAADATNATGAIKAIDDALRTVAENRATLGATLNRFDFNVNNLKSQESTMASSASQ     245
F528/94           --------AAGALAAISSIDKALKTVAGDRADLGATLNRLNFNVENLNNQATNMASSASQ     230
bcf_08380         ---ITGTDGTTADEAIETIDTMIDAIAGNRATLGATLNRLQYNAENLNNQSTAMSSSASQ     226
RIVM_Bc126        ------------------------------------------------------------     249
MHI86             VAKSSDDSAANPLQAIRAIDAALKTIADNRATLGATLNRLDFNVNNLKSQQSSMASAASQ     404
F4429/71          VAKSSDDSAANPLQAIRAIDAALKTIADNRATLGATLNRLDFNVNNLKSQQSSMASAASQ     404
RIVM_Bc964        ----TTDLKANASGAIASIDTALKNIASNRATLGATLNRLDFNVNNLKSQSSSMASAASQ     322
F3162/04_(D8)     AKTAG--TTEKSLVAVDKIDEALKTIADNRATLGATLNRLDFNVNNLKSQSASMASAASQ     423
MHI226            -TATAAADKVDALAAVEAIDKALTTVADNRATLGATLNRLDFNVNNLKSQSSSMASAASQ     321
NVH_0075-95       PATGGAQGSANSLGAITKIDAALKTVADNRATLGATLNRLDFNVNNLKSQASSMAAAASQ     331
WSBS10035         FATAADESAANPLQAIKAIDAAIKNIADNRATLGATLNRLDFNVNNLKSQASSMASAASQ     403
RIVM_Bc90         FATAADESAANPLQAIKAIDAAIKNIADNRATLGATLNRLDFNVNNLKSQASSMASAASQ     403
7/27/S            AKEALNPANAMPLDAVKQIDAALKTVADNRATLGATLNRLDFNVNNLKSQSSAMAASASQ     452
IP5832            -----KVTVANPNAAVAAIDSALENIASNRATLGATLNRLDFNVNNLKSQSSSMASAASQ     317


14294-3_(M6)      IEDADMAKEMSEMTKFKILNEAGISMLSQANQTPQMVSKLLQ      364        -> 39.00 kDa
SDA_KA96          IEDADMAKEMSEMTKFKILNEAGISMLSQANQTPQMVSKLLQ      457        -> 48.59 kDa
INRA_A3           IEDADMAKEMSEMTKFKILNEAGISMLSQANQTPQMVSKLLQ      371        -> 40.42 kDa
INRA_C3           IEDADMAKEMSEMTKFKILNEAGISMLSQANQTPQMVSKLLQ      393        -> 42.43 kDa
6/27/S            IEDADMAKEMSEMTKFKILNEAGISMLSQANQTPQMVSKLLQ      287        -> 31.02 kDa
F3175-03_(D7)     ------------------------------------------      142        -> 15.23 kDa
RIVM_Bc934        IEDADMAKEMSEMTKFKILNEAGISMLSQANQTPQMVSKLLQ      287        -> 31.02 kDa
F528/94           IEDADMAKEMSEMTKFKILNEAGISMLSQANQTPQMVSKLLQ      272        -> 29.27 kDa
bcf_08380         IEDADMAKEMSEMTKFKILNEAGISMLSQANQTPQMVSKLLQ      268        -> 28.74 kDa
RIVM_Bc126        ------------------------------------------      249        -> 26.71 kDa
MHI86             VEDADMAKEMSEMTKFKILNEAGISMLSQANQTPQMVSKLLQ      446        -> 47.56 kDa
F4429/71          VEDADMAKEMSEMTKFKILNEAGISMLSQANQTPQMVSKLLQ      446        -> 47.56 kDa
RIVM_Bc964        IEDADMAKEMSEMTKFKILNEAGISMLSQANQTPQMVSKLLQ      364        -> 39.26 kDa
F3162/04_(D8)     IEDADMAKEMSEMTKFKILNEAGISMLSQANQTPQMVSKLLQ      465        -> 49.85 kDa
MHI226            IEDADMAKEMSEMTKFKILNEAGISMLSQANQTPQMVSKLLQ      363        -> 38.38 kDa
NVH_0075-95       VEDADMAKEMSEMTKFKILNEAGISMLSQANQTPQMVSKLLQ      373        -> 39.96 kDa
WSBS10035         VEDADMAKEMSEMTKFKILNEAGISMLSQANQTPQMVSKLLQ      445        -> 47.50 kDa
RIVM_Bc90         VEDADMAKEMSEMTKFKILNEAGISMLSQANQTPQMVSKLLQ      445        -> 47.50 kDa
7/27/S            IEDADMAKEMSEMTKFKILNEAGISMLSQANQTPQMVSKLLQ      494        -> 52.57 kDa
IP5832            IEDADMAKEMSEMTKFKILNEAGISMLSQANQTPQMVSKLLQ      359        -> 38.18 kDa
```

D. recombinant flagellin proteins

- rFla full length without putative N-terminal signal peptide for secretion
  258 aa, 27.2 kDa, strep-tag, linker, mAb 1A11 epitope

MASWSHPQFEKGAETAVPNSSSVPRINSAADDAAGLAIATRMRAQESGLNVASRNTQDGISLIQTADSALGTVSNILHRMRDLAVQSANGSNGE
TDRDALDGEYQQLITQIGYIADNTEFNGTNLLKADTTISIKSTDKSKASTIDIETKAVDGSGLGSITGTDGTTADEAIETIDTMIDAIAGNRAT
LGATLNRLQYNAENLNNQSTAMSSSASQIEDADMAKEMSEMTKFKILNEAGISMLSQANQTPQMVSKLLQ

- rFla1, 102 aa, 10.6 kDa

MASWSHPQFEKGAETAVPNSSSVPRINSAADDAAGLAIATRMRAQESGLNVASRNTQDGISLIQTADSALGTVSNILHRMRDLAVQSANGSNGE
TDRDALDG

- rFla2, 125 aa, 13.28 kDa

MASWSHPQFEKGAETAVPNSSSVPQESGLNVASRNTQDGISLIQTADSALGTVSNILHRMRDLAVQSANGSNGETDRDALDGEYQQLITQIGYI
ADNTEFNGTNLLKADTTISIKSTDKSKASTI

- rFla3, 103 aa, 10.8 kDa

MASWSHPQFEKGAETAVPNSSSVPEYQQLITQIGYIADNTEFNGTNLLKADTTISIKSTDKSKASTIDIETKAVDGSGLGSITGTDGTTADEAI
ETIDTMIDA

- rFla4, 131 aa, 13.77 kDa

MASWSHPQFEKGAETAVPNSSSVPDIETKAVDGSGLGSITGTDGTTADEAIETIDTMIDAIAGNRATLGATLNRLQYNAENLNNQSTAMSSSAS
QIEDADMAKEMSEMTKFKILNEAGISMLSQANQTPQM

- rFla5, 101 aa, 10.9 kDa

MASWSHPQFEKGAETAVPNSSSVPIAGNRATLGATLNRLQYNAENLNNQSTAMSSSASQIEDADMAKEMSEMTKFKILNEAGISMLSQANQTPQ
MVSKLLQ

E. flagellin promoter alignment of 21 strains

```
F3175/03_(D7)     AATATCACCTTTTCGGTAAACAAAGATAATCTAATATTAGAGTTTTTTTTTAAAGTTTGC     60
RIVM_Bc_126       AATATCACCTTTTCGGTAAACAAAGATAATCTAATATTAGAGTTTTTTCGTAAAGTTTGC     60
F528/94           AATATCACCTTTTCGGTAAACAAAGATAATCTAATATTAGAGTTTTTTTTTAAAGTTTGC     60
6/27/S            AATATCACCTTTTCGGTAAACAAAGATAATCTAATATTAGAGTTTTTTTTTAAAGTTTGC     60
RIVM_Bc_934       AATATCACCTTTTCGGTAAACAAAGATAATCTAATATTAGAGTTTTTTTTTAAAGTTTGC     60
MHI_226           AATATCACCTTTTCGGTAAACAAAGATAATCTAATATTAGAGTTTTTTTTTAAAGTTTGC     60
bcf_08380         AATATCACCTTTTCGGTAAACAAAGATAATCTAATATTAGAGTTTTTTTTTAAAGTTTGC     60
F837/76_2         AATATCACCTTTTCGGTAAACAAAGATAATCTAATATTAGAGTTTTTTTTTAAAGTTTGC     60
14294-3_(M6)      AATATCACCTTTTCGGTAAACAAAGATAATCTAATATTAGAGTTTTTTTTTAAAGTTTGC     60
SDA_KA96          AATATCACCTTTTCGGTAAACAAAGATAATCTAATATTAGAGTTTTTTTTTAAAGTTTGC     60
INRA_C3           AATATCACCTTTTCGGTAAACAAAGATAATCTAATATTAGAGTTTTTTTTTAAAGTTTGC     60
INRA_A3           AATATCACCTTTTCGGTAAACAAAGATAATCTAATATTAGAGTTTTTTTTTAAAGTTTGC     60
RIVM_Bc_964       AATATCACCTTTTCGGTAAACAAAGATAATCTAATATTAGAGTTTTTTTTTAAAGTTTGC     60
IP5832            AATATCACCTTTTCGGTAAACAAAGATAATCTAATATTAGAGTTTTTTTTTAAAGTTTGC     60
MHI_86            AATATCACCTTTTCGGTAAACAAAGATAATCTAATATTAGAGTTTTTTCGTAAAGTTTGC     60
F4429/71          AATATCACCTTTTCGGTAAACAAAGATAATCTAATATTAGAGTTTTTTCGTAAAGTTTGC     60
F3162/04_(D8)     AATATCACCTTTTCGGTAAACAAAGATAATCTAATATTAGAGTTTTTTCGTAAAGTTTGC     60
WSBC_10035        AATATCACCTTTTCGGTAAACAAAGATAATCTAATATTAGAGTTTTTTCGTAAAGTTTGC     60
RIVM_Bc_90        AATATCACCTTTTCGGTAAACAAAGATAATCTAATATTAGAGTTTTTTCGTAAAGTTTGC     60
7/27/S            AATATCACCTTTTCGGTAAACAAAGATAATCTAATATTAGAGTTTTTTCGTAAAGTTTGC     60
NVH_0075-95       AATATCACCTTTTCGGTAAACAAAGATAATCTAATATTAGAGTTTTTTCGTAAAGTTTGC     60
                  ************************************************  **********

F3175/03_(D7)     AATAATCCTTTTGATAA-----------------------TATTGTAAATTATCATTTT     96
RIVM_Bc_126       AATAATCCTTTGCAAT-----------AAAAAAACGACTAGATTGTTAAACTGTTATTTT     109
F528/94           AATAATCCTTT-TAATAATATTGTAATAAAAAAACGACTAAATTGTTAAACTGTTATTTT     119
6/27/S            AATAATCCTTT-TAATAATTATGTAATAAAAAAATGAATATATTGTTAAACTGTTATTTT     119
RIVM_Bc_934       AATAATCCTTT-TAATAATTATGTAATAAAAAAATGAATATATTGTTAAACTGTTATTTT     119
MHI_226           AATAATCCTTT-TAATAATATTTTAATAAAAAAATGAATATATTGTTAAACTGTTATTTT     119
bcf_08380         AATAATCCTTT-TAATAATATTGTAATAAAAAAATGAATATATTGTTAAACTGTTATTTT     119
F837/76_2         AATAATCCTTT-TAATAATATTGTAATAAAAAAATGAATATATTGTTAAACTGTTATTTT     119
14294-3_(M6)      AATAATCCTTT-TAATAATATTGTAATAAAAAAATGAATATATTGTTAAACTGTTATTTT     119
SDA_KA96          AATAATCCTTT-TAATAATATTGTAATAAAAAAATGAATATATTGTTAAACTGTTATTTT     119
INRA_C3           AATAATCCTTT-TAATAATTATGTAATAAAAAAATGAATATATTGTTAAACTGTTATTTT     119
INRA_A3           AATAATCCTTT-TAATAATTATGTAATAAAAAAATGGATATATTGTTAAACTGTTATTTT     119
RIVM_Bc_964       AATAATCCTTT-TAATAATTATGTAATAAAAAAATGAATATATTGTTAAACTGTTATTTT     119
IP5832            AATAATCCTTT-TAATAATTATGTAATAAAAAAATGAATATATTGTTAAACTGTTATTTT     119
MHI_86            AATAATCCTTTGCAATTATATTGTAATAAAAAAACGACTAAATTGTTAAACTGTTATTTT     120
F4429/71          AATAATCCTTTGCAATTATATTGTAATAAAAAAACGACTAAATTGTTAAACTGTTATTTT     120
F3162/04_(D8)     AATAATCCTTTGCAATTATATTGTAATAAAAAAACGACTAAATTGTTAAACTGTTATTTT     120
WSBC_10035        AATAATCCTTTGCAATTATATTGTAATAAAAAAACGACTAAATTGTTAAACTGTTATTTT     120
RIVM_Bc_90        AATAATCCTTTGCAATTATATTGTAATAAAAAAACGACTAAATTGTTAAACTGTTATTTT     120
7/27/S            AATAATCCTTTGCAATTATATTGTAATAAAAAAACGACTAAATTGTTAAACTGTTATTTT     120
NVH_0075-95       AATAATCCTTTGCAATTATATTGTAATAAAAAAACGACTAAATTGTTAAACTGTTATTTT     120
                  ***********  *                             *  **** * * *****
```

```
F3175/03_(D7)       TTTATATTCATTAATTACAATTATTCGTTGACACGATATATACCAAGGATTACAATTAAT      156
RIVM_Bc_126         TTTATATTTGTTAATTACAATTATTCGTTGACACGATATACACCAAGGATTACAATTAAT      169
F528/94             TTTATATTTATTAATTCGAATTTTTAGTTGACACCCACTATGACAGGGATTACAATAAAT      179
6/27/S              TTTATATTTATTAATTCGAATTTTTAGTTGACACCCGCTATGGCAGGGATTACAATAAAT      179
RIVM_Bc_934         TTTATATTTATTAATTCGAATTTTTAGTTGACACCCGCTATGGCAGGGATTACAATAAAT      179
MHI_226             TTTATATTTATTAATTCGAATTTTTAGTTGACACCCGCTATGACAGGGATTACAATAAAT      179
bcf_08380           TTTATATTTATTAATTCGAATTTTTAGTTGACACCCGCTATGACAGGGATTACAATAAAT      179
F837/76_2           TTTATATTTATTAATTCGAATTTTTAGTTGACACCCGCTATGACAGGGATTACAATAAAT      179
14294-3_(M6)        TTTATATTTATTAATTCGAATTTTTAGTTGACACCCGCTATGACAGGGATTACAATAAAT      179
SDA_KA96            TTTATATTTATTAATTCGAATTTTTAGTTGACACCCGCTATGACAGGGATTACAATAAAT      179
INRA_C3             TTTATATTTATTAATTCGAATTTTTAGTTGACACCCGCTATGACAGGGATTACAATAAAT      179
INRA_A3             TTTATATTTATTAATTCGAATTTTTAGTTGACACCCGTTGTGGCAAGGATTACAATAAAT      179
RIVM_Bc_964         TTTATATTTATTAATTCGAATTTTTAGTTGACACCCGTTGTAGCAAGGATTACAATAAAT      179
IP5832              TTTATATTTATTAATTCGAATTTTTAGTTGACACCTGTTGTAGCAAGGATTACAATAAAT      179
MHI_86              TTTATATTTATTAATTCAAATTTTCAGTTGACACCCATTATAGCAAGGCTTACAATAAAT      180
F4429/71            TTTATATTTATTAATTCAAATTTTCAGTTGACACCCATTATAGCAAGGCTTACAATAAAT      180
F3162/04_(D8)       TTTATATTTATTAATTCAAATTTTCAGTTGACACCCATTATAGCAAGGCTTACAATAAAT      180
WSBC_10035          TTTATATTTATTAATTCAAATTTTCAGTTGACACCCATTATAGCAAGGCTTACAATAAAT      180
RIVM_Bc_90          TTTATATTTATTAATTCAAATTTTCAGTTGACACCCATTATAGCAAGGCTTACAATAAAT      180
7/27/S              TTTATATTTATTAATTCAAATTTTCAGTTGACACCCATTATAGCAAGGCTTACAATAAAT      180
NVH_0075-95         TTTATATTTATTAATTCAAATTTTCAGTTGACACTTGATATAGCAAGGCTTAAAATAAAT      180
                    ********  ******  **** *  ********    *    ** ** *** *** ***

F3175/03_(D7)       CTCGAAATTGATTTTTTTGCATATTTACGTTTAACAATTAACAGAAAAATCAATATAAAA      216
RIVM_Bc_126         ATT----------------------ACTTTTTTGCAATTTGCAGAATAGTCAATATAAAA      207
F528/94             CTCGAAATTGATTTTTTCGCATATTACCGTTTTTCAATTTGCAGAAAAGTCAATATAAAA      239
6/27/S              CTCGAAATTGATTTTTTCGCATATTACCGTTTTTCAATTTGCAGAAAAGTCAATATAAAA      239
RIVM_Bc_934         CTCGAAATTGATTTTTTCGCATATTACCGTTTTTCAATTTGCAGAAAAGTCAATATAAAA      239
MHI_226             CTCGAAATTGATTTTTTCGCATATTACCGTTTTTCAATTTGCAGAAAAGTCAATATAAAA      239
bcf_08380           CTCGAAATTGATTTTTTTGCATATTACCGTTTTTCAATTTGCAGAAAAGTCAATATAAAA      239
F837/76_2           CTCGAAATTGATTTTTTTGCATATTACCGTTTTTCAATTTGCAGAAAAGTCAATATAAAA      239
14294-3_(M6)        CTCGAAATTGATTTTTTCGCATATTACCGTTTTTCAATTTGCAGAAAAGTCAATATAAAA      239
SDA_KA96            CTCGAAATTGATTTTTTCGCATATTATCGTTTTTCAATTTGCAGAAAAGTCAATATAAAA      239
INRA_C3             CTCGAAATTGATTTTTTCGCATATTACCGTTTTTCAATTTGCAGAAAAGTCAATATAAAA      239
INRA_A3             CTCGAAATTGATTTTTTCGCATATTACCGTTTTTCAATTTGCAGAAAAGTCAATATAAAA      239
RIVM_Bc_964         CTCGAAATTGATTTTTTCGCATATTACCGTTTTTCAATTTGCAGAAAAGTCAATATAAAA      239
IP5832              CTCGAAATTGATTTTTTCGCATATTACCGTTTTTCAATTTGCAGAAAAGTCAATATAAAA      239
MHI_86              -TCGAAATTGTTTTTTTTGCATATTACCGTTTTTCAATTTGCAGAAAACTCAATATAAAA      239
F4429/71            -TCGAAATTGTTTTTTTTGCATATTACCGTTTTTCAATTTGCAGAAAACTCAATATAAAA      239
F3162/04_(D8)       -TCGAAATTGTTTTTTTTGCATATTACCGTTTTTCAATTTGCAGAAAACTCAATATAAAA      239
WSBC_10035          -TCGAAATTGTTTTTTTTGCATATTACCGTTTTTCAATTTGCAGAAAACTCAATATAAAA      239
RIVM_Bc_90          -TCGAAATTGTTTTTTTTGCATATTACCGTTTTTCAATTTGCAGAAAACTCAATATAAAA      239
7/27/S              -TCGAAATTGTTTTTTTTGCATATTACCGTTTTTCAATTTGCAGAAAAATCAATATAAAA      239
NVH_0075-95         -TCGAAATCGATTTTTTCGCATATTACCGTTTTTCAATTTGCAGAAAAGTCAATATAAAA      239
                     *                           ***  *****  ***** * ***********

F3175/03_(D7)       AAATGGGGGTTTCTTAA   233
RIVM_Bc_126         AAATGGGGGTTCTTAAC   224
F528/94             AAATGGGGGGTTCTTAAC  256
6/27/S              AAATGGGGGTTCTTAAC   256
RIVM_Bc_934         AAATGGGGGTTCTTAAC   256
MHI_226             AAATGGGGGTTCTTAAT   256
bcf_08380           AAATGGGGGTTCTTAAC   256
F837/76_2           AAATGGGGGTTCTTAAC   256
14294-3_(M6)        AAATGGGGGTTCTTAAC   256
SDA_KA96            AAATGGGGGTTCTTAAC   256
INRA_C3             AAATGGGGGGTTTCTTAA  256
INRA_A3             AAATGGGGGTTTCTTAA   256
RIVM_Bc_964         AAATGGGGGTTTCTTAA   256
IP5832              AAATGGGGGTTTCTTAA   256
MHI_86              AAATGGGGGTTCTTAAC   256
F4429/71            AAATGGGGGTTCTTAAC   256
F3162/04_(D8)       AAATGGGGGTTCTTAAC   256
WSBC_10035          AAATGGGGGTTCTTAAC   256
RIVM_Bc_90          AAATGGGGGTTCTTAAC   256
```

Fig S2. Sequences and multiple sequence alignments (CLUSTAL O 1.2.4) of *B. cereus* flagellin. A. Comparison of bcf_08380 (strain F837/76) and the three verified flagellin-encoding genes of strain ATCC 14579. **B.** Alignment of the corresponding protein sequences. The epitope most likely recognized by mAb 1A11 is marked in green. **C.** Alignment of the bcf_08380 gene product and its homologues of 19 enteropathogenic and apathogenic *B. cereus* strains. bcf_08380 is highlighted in bold. The mAb 1A11 epitope is shown in green. Strains INRA C3 and F3175-03_(D7) are underlined. Amino acid substitutions in the N-terminal region are highlighted (red: only strain F3175-03_(D7), yellow: only INRA C3, pink: only few strains including INRA C3 or F3175-03_(D7)). **D.** Sequences of the recombinant flagellin proteins (rFla) after cloning in the pASK-IBA5+ vector. The putative N-terminal signal peptide for secretion was replaced by a strep-tag and a linker sequence. **E.** Alignment of the bcf_08380 promoter region and its homologues of 20 *B. cereus* strains. bcf_08380 is highlighted in bold. Green: -35 region, orange: -10 region, yellow: +1 according to [1]. Red: Deviations to these sequences in two strains.

1. Ankarloo J, Zhang MY, Lövgren A. Regulatory sequences of two flagellin genes in *Bacillus thuringiensis* subsp. *alesti*. Microbiology (Reading, Engl.). 1996;142(2):315-320.
