# Supplementary material for: Characterization of strain-specific Bacillus cereus swimming motility and flagella by means of specific antibodies
Source: PLoS One. 2022 Mar 17;17(3):e0265425. doi: 10.1371/journal.pone.0265425 (PMC8929632; doi:10.1371/journal.pone.0265425)
Supplement: S3 Fig — A. Highly specific indirect EIAs with mAb 1A11. B. Indirect EIAs with rabbit antiserum # 5321. C. Highly specific sandwich EIAs. Bt: B. thuringiensis, Bw: B. weihenstephanensis, Bps: B. pseudomycoides, Bs: B. subtilis, Bl: B. licheniformis, Ba: B. amyloliquefaciens, Bpu: B. pumilus, Lm: L. monocytogenes. B. cereus strain F837/76 is shown for comparison. Results indicate means and standard deviations of two biological with three technical replicates for each strain. (PDF) [file pone.0265425.s003.pdf]

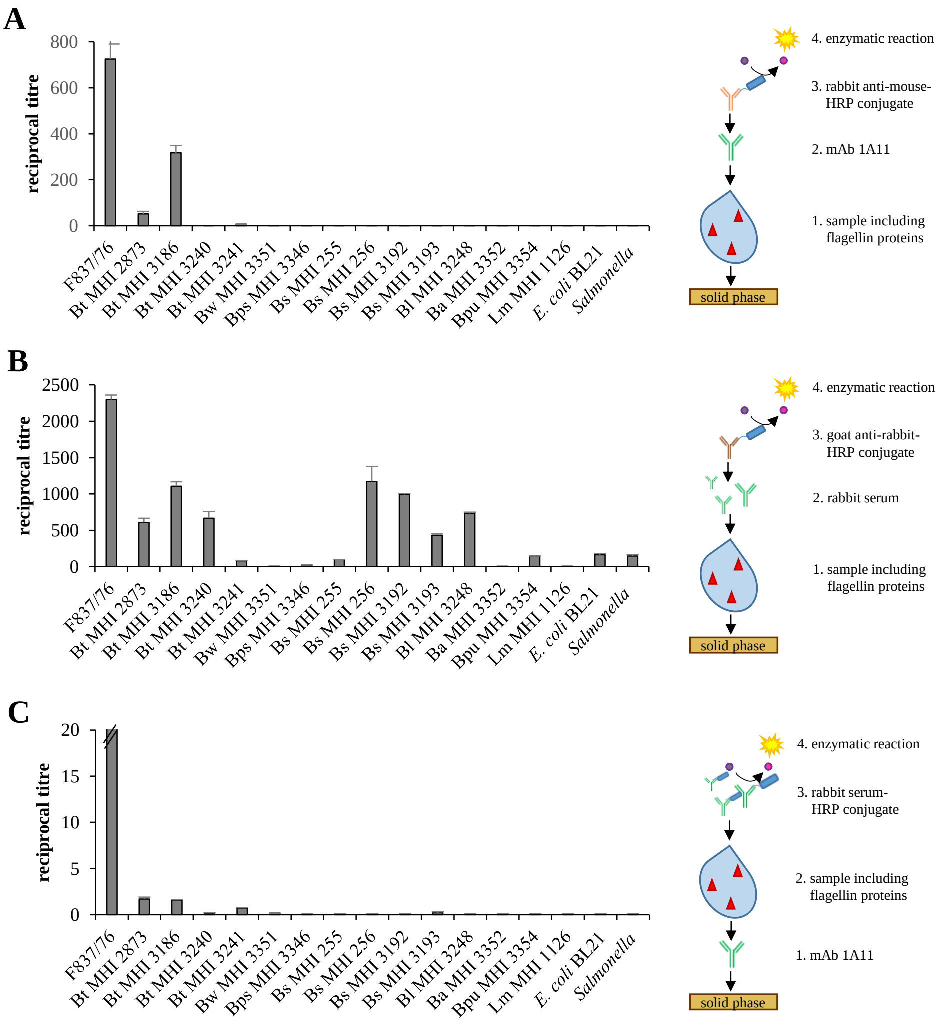

Fig S3. Negative controls show the specificity of the established EIAs. A. Highly specific indirect EIAs with mAb 1A11. **B.** Indirect EIAs with rabbit antiserum # 5321. **C.** Highly specific sandwich EIAs. Bt: *B. thuringiensis*, Bw: *B. weihenstephanensis*, Bps: *B. pseudomycoides*, Bs: *B. subtilis*, Bl: *B. licheniformis*, Ba: *B. amyloliquefaciens*, Bpu: *B. pumilus*, Lm: *L. monocytogenes*. *B. cereus* strain F837/76 is shown for comparison. Results indicate means and standard deviations of two biological with three technical replicates for each strain.
